# Incidence, Clinical Characteristics and Management of Inflammatory Bowel Disease in Spain: Large-Scale Epidemiological Study

**DOI:** 10.3390/jcm10132885

**Published:** 2021-06-29

**Authors:** María Chaparro, Ana Garre, Andrea Núñez Ortiz, María Teresa Diz-Lois Palomares, Cristina Rodríguez, Sabino Riestra, Milagros Vela, José Manuel Benítez, Estela Fernández Salgado, Eugenia Sánchez Rodríguez, Vicent Hernández, Rocío Ferreiro-Iglesias, Ángel Ponferrada Díaz, Jesús Barrio, José María Huguet, Beatriz Sicilia, María Dolores Martín-Arranz, Xavier Calvet, Daniel Ginard, Inmaculada Alonso-Abreu, Luis Fernández-Salazar, Pilar Varela Trastoy, Montserrat Rivero, Isabel Vera-Mendoza, Pablo Vega, Pablo Navarro, Mónica Sierra, José Luis Cabriada, Mariam Aguas, Raquel Vicente, Mercè Navarro-Llavat, Ana Echarri, Fernando Gomollón, Elena Guerra del Río, Concepción Piñero, María José Casanova, Katerina Spicakova, Jone Ortiz de Zarate, Emilio Torrella Cortés, Ana Gutiérrez, Horacio Alonso-Galán, Álvaro Hernández-Martínez, José Miguel Marrero, Rufo Lorente Poyatos, Margalida Calafat, Lidia Martí Romero, Pilar Robledo, Orencio Bosch, Nuria Jiménez, María Esteve Comas, José María Duque, Ana María Fuentes Coronel, Manuela Josefa Sampedro, Eva Sesé Abizanda, Belén Herreros Martínez, Liliana Pozzati, Hipólito Fernández Rosáenz, Belén Crespo Suarez, Pilar López Serrano, Alfredo J. Lucendo, Margarita Muñoz Vicente, Fernando Bermejo, José Joaquín Ramírez Palanca, Margarita Menacho, Amalia Carmona, Raquel Camargo, Sandra Torra Alsina, Nuria Maroto, Juan Nerín de la Puerta, Elena Castro, Ignacio Marín-Jiménez, Belén Botella, Amparo Sapiña, Noelia Cruz, José Luis F. Forcelledo, Abdel Bouhmidi, Carlos Castaño-Milla, Verónica Opio, Isabel Nicolás, Marcos Kutz, Alfredo Abraldes Bechiarelli, Jordi Gordillo, Yolanda Ber, Yolanda Torres Domínguez, María Teresa Novella Durán, Silvia Rodríguez Mondéjar, Francisco J. Martínez-Cerezo, Lilyan Kolle, Miriam Sabat, Cesar Ledezma, Eduardo Iyo, Óscar Roncero, Rebeca Irisarri, Laia Lluis, Isabel Blázquez Gómez, Eva María Zapata, María José Alcalá, Cristina Martínez Pascual, María Montealegre, Laura Mata, Ana Monrobel, Alejandro Hernández Camba, Luis Hernández, María Tejada, Alberto Mir, María Luisa Galve, Marta Soler, Daniel Hervías, José Antonio Gómez-Valero, Manuel Barreiro-de Acosta, Fernando Rodríguez-Artalejo, Esther García-Esquinas, Javier P. Gisbert, on behalf of the EpidemIBD study group of GETECCU

**Affiliations:** 1 Department of Gastroenterology, Hospital Universitario de La Princesa, Instituto de Investigación Sanitaria Princesa (IIS-IP), Universidad Autónoma de Madrid, and Centro de Investigación Biomédica en Red de Enfermedades Hepáticas y Digestivas (CIBERehd), 28006 Madrid, Spain; anagarre.laprincesa@gmail.com (A.G.); mjcasanova.g@gmail.com (M.J.C.); javier.p.gisbert@gmail.com (J.P.G.); 2 Department of Gastroenterology, Hospital Universitario Virgen del Rocío, 41013 Sevilla, Spain; andreanuor@gmail.com; 3 Department of Gastroenterology, Hospital Universitario A Coruña, 15006 A Coruña, Spain; maitedlp@yahoo.es; 4 Department of Gastroenterology, Complejo Hospitalario de Navarra, 31008 Pamplona, Spain; cristina.rodriguez.gutierrez@cfnavarra.es; 5 Department of Gastroenterology, Hospital Universitario Central de Asturias and ISPA, 33011 Oviedo, Spain; sriestram7@gmail.com; 6 Department of Gastroenterology, Complejo Hospitalario Universitario Ntra. Sra. de Candelaria, 38010 Santa Cruz de Tenerife, Spain; Milvelillas@yahoo.es; 7 Department of Gastroenterology, Hospital Universitario Reina Sofía and IMIBIC, 14004 Córdoba, Spain; jmbeni83@hotmail.com; 8 Department of Gastroenterology, Complexo Hospitalario Universitario de Pontevedra, Instituto de Investigación Sanitaria Galicia Sur, 36071 Pontevedra, Spain; estela.fernandez.salgado@sergas.es; 9 Department of Gastroenterology, Hospital Ramón y Cajal, 28034 Madrid, Spain; eugenia.sanchez.rodriguez@gmail.com; 10 Department of Gastroenterology, Hospital Álvaro Cunqueiro, Estrutura Organizativa de Xestión Integrada de Vigo, 36213 Vigo, Spain; vicent.Hernandez.Ramirez@sergas.es; 11 Department of Gastroenterology, Complexo Hospitalario Universitario de Santiago, 15706 Santiago de Compostela, Spain; rocioferstg@hotmail.com (R.F.-I.); manubarreiro@hotmail.com (M.B.-d.A.); 12 Department of Gastroenterology, Hospital Universitario Infanta Leonor, 28031 Madrid, Spain; angelponmedicina@yahoo.es; 13 Department of Gastroenterology, Hospital Universitario Rio Hortega, 47012 Valladolid, Spain; jbarrioa95@gmail.com; 14 Department of Gastroenterology, Consorcio Hospital General Universitario de Valencia, 46014 Valencia, Spain; josemahuguet@gmail.com; 15 Department of Gastroenterology, Hospital Universitario de Burgos, 09006 Burgos, Spain; bsicilia4@gmail.com; 16 Department of Gastroenterology, Hospital Universitario La Paz, 28046 Madrid, Spain; mmartinarranz@gmail.com; 17 Servei de Malalties Digestives, Hospital de Sabadell, Institut Universitari Parc Taulí, Universitat Autònoma de Barcelona, CIBEREHD—Instituto de Salud Carlos III, Parc Taulí, 1, 08208 Sabadell, Barcelona, Spain; xcalvet@tauli.cat; 18 Department of Gastroenterology, Hospital Universitari Son Espases, 07120 Palma de Mallorca, Spain; daniel.ginard@gmail.com; 19 Department of Gastroenterology, Hospital Universitario de Canarias (H.U.C.), 38320 Santa Cruz de Tenerife, Spain; inmaculadaalonsoabreu@gmail.com; 20 Department of Gastroenterology, Hospital Clínico Universitario de Valladolid, 47003 Valladolid, Spain; luisfernsal@gmail.com; 21 Department of Gastroenterology, Hospital de Cabueñes, 33394 Gijón, Spain; trastoy@hotmail.com; 22 Department of Gastroenterology, Hospital Universitario Marqués de Valdecilla and IDIVAL, 39008 Santander, Spain; digrtm@humv.es; 23 Department of Gastroenterology, Hospital Universitario Puerta de Hierro Majadahonda, 28222 Madrid, Spain; isabel.veramendoza@gmail.com; 24 Department of Gastroenterology, Complexo Hospitalario Universitario de Ourense, 32005 Ourense, Spain; PABLO.VEGA.VILLAAMIL@sergas.es; 25 Department of Gastroenterology, Hospital Clínico Universitario de Valencia, Universitat de València, 46010 Valencia, Spain; pnavarrocortes@gmail.com; 26 Department of Gastroenterology, Complejo Asistencial Universitario de León, 24001 León, Spain; msierra.ausin@gmail.com; 27 Department of Gastroenterology, Hospital de Galdakao-Usansolo, Galdakao, 48960 Vizcaya, Spain; joseluis.cabriadanuno@osakidetza.eus; 28 Department of Gastroenterology, Hospital Universitari i Politecnic La Fe and CIBERehd, 46026 Valencia, Spain; aguas_mar@gva.es; 29 Department of Gastroenterology, Hospital Universitario Miguel Servet, 50009 Zaragoza, Spain; raquelvicentelidon@gmail.com; 30 Department of Gastroenterology, Hospital de Sant Joan Despí Moisès Broggi, 08970 Barcelona, Spain; mnavarrollavat@gmail.com; 31 Department of Gastroenterology, Complejo Hospitalario Universitario de Ferrol, 15405 A Coruña, Spain; ana.echarri.piudo@sergas.es; 32 Department of Gastroenterology, Hospital Clínico Universitario “Lozano Blesa”, IIS Aragón and CIBERehd, 50009 Zaragoza, Spain; fgomollon@gmail.com; 33 Department of Gastroenterology, Hospital Universitario de Gran Canaria Dr. Negrín, 35010 Las Palmas, Spain; elenoti@hotmail.com; 34 Department of Gastroenterology, Hospital Universitario de Salamanca, 37007 Salamanca, Spain; conxipi@hotmail.com; 35 Department of Gastroenterology, Hospital Universitario de Araba (sede Txagorritxu y sede Santiago), 01009 Álava, Spain; katerina.spicakova-@osakidetza.eus; 36 Department of Gastroenterology, Hospital Universitario de Basurto, 48013 Bilbao, Spain; jone.ortizdezaratesagastagoitia@osakidetza.net; 37 Department of Gastroenterology, Hospital General Universitario J.M. Morales Meseguer, 30008 Murcia, Spain; emilioa.torrella@gmail.com; 38 Department of Gastroenterology, Hospital General Universitario de Alicante and CIBERehd, 03010 Alicante, Spain; gutierrez_anacas@gva.es; 39 Department of Gastroenterology, Hospital Universitario Donostia-Donostia Unibertsitate Ospitalea, Guipuzkoa and Organizacion Sanitaria Integrada Tolosaldea, Clínica Santa María de la Asunción, 20014 Guipúzcoa, Spain; horacio.alonsogalan@osakidetza.eus; 40 Department of Gastroenterology, Complejo Hospitalario de Especialidades Torrecárdenas, 04009 Almería, Spain; alvarohernandezm68@gmail.com; 41 Department of Gastroenterology, Hospital Universitario Insular de Gran Canaria, 35016 Las Palmas, Spain; marreromonroy@hotmail.com; 42 Department of Gastroenterology, Hospital General Universitario de Ciudad Real, 13005 Ciudad Real, Spain; rufolorente@hotmail.com; 43 Department of Gastroenterology, Hospital Son Llatzer, 07198 Palma de Mallorca, Spain; margalidasard.calafat@gmail.com; 44 Department of Gastroenterology, Hospital Francesc De Borja de Gandía, 46702 Valencia, Spain; lidia.marti.romero@gmail.com; 45 Department of Gastroenterology, Hospital Universitario de Cáceres, 10004 Cáceres, Spain; probledoa@gmail.com; 46 Department of Gastroenterology, Hospital Universitario Fundación Jiménez Díaz, 28040 Madrid, Spain; OBosch@quironsalud.es; 47 Department of Gastroenterology, Hospital General Universitario de Elche, 03203 Alicante, Spain; nurjime@gmail.com; 48 Department of Gastroenterology, Hospital Universitari Mutua Terrasa, 08221 Terrassa, Spain; mestevecomas@telefonica.net; 49 Department of Gastroenterology, Hospital San Agustín, 33401 Avilés, Spain; josemaria.duque@sespa.es; 50 Department of Gastroenterology, Hospital Virgen de La Concha, Complejo Asistencial de Zamora, 49022 Zamora, Spain; amfcoronel@gmail.com; 51 Department of Gastroenterology, CSDM, Hospital de Mataró, 08304 Barcelona, Spain; msampedro@csdm.cat; 52 Department of Gastroenterology, Hospital Universitario Arnau de Vilanova, 25198 Lérida, Spain; eseseabi@gmail.com; 53 Department of Gastroenterology, Hospital De Villajoyosa, 03570 Alicante, Spain; belherreros@gmail.com; 54 Department of Gastroenterology, Hospital de Mérida, 06800 Mérida, Spain; santelmo0054@hotmail.com; 55 Department of Gastroenterology, Hospital San Pedro, 26006 Logroño, Spain; hfrosaenz@riojasalud.es; 56 Department of Gastroenterology, Hospital da Costa (EOXI Lugo-Cervo-Monforte), 27880 Lugo, Spain; belen.Crespo.Suarez@sergas.es; 57 Department of Gastroenterology, Hospital Universitario Fundación Alcorcón, 28922 Madrid, Spain; plopez@fhalcorcon.es; 58 Department of Gastroenterology, Hospital General de Tomelloso, 13700 Tomelloso, Spain; ajlucendo@hotmail.com; 59 Instituto de Investigación Sanitaria Princesa (IIS-IP), 28006 Madrid, Spain; 60 Centro de Investigación Biomédica en Red de Enfermedades Hepáticas y Digestivas (CIBERehd), 28006 Madrid, Spain; 61 Department of Gastroenterology, Hospital General Universitario de Castellón, 12004 Castellón, Spain; munyoz_marvice@yahoo.es; 62 Department of Gastroenterology, Hospital Universitario de Fuenlabrada and Instituto de Investigación Sanitaria Hospital La Paz (IdiPaz), 28942 Madrid, Spain; fbermejos1@gmail.com; 63 Department of Gastroenterology, Hospital Lluis Alcanyis, Xátiva, 46800 Valencia, Spain; joramirezpa@gmail.com; 64 Department of Gastroenterology, Hospital Joan XXIII, 43005 Tarragona, Spain; mmenacho.hj23.ics@gencat.cat; 65 Department of Gastroenterology, Hospital Povisa, 36211 Pontevedra, Spain; amiccar@hotmail.com; 66 Department of Gastroenterology, Complejo Hospitalario de Especialidades Virgen de la Victoria, 29010 Málaga, Spain; raquelcamero@hotmail.com; 67 Department of Gastroenterology, Parc Sanitari Sant Joan de Déu, 08830 Barcelona, Spain; sandra.torra@pssjd.org; 68 Department of Gastroenterology, Hospital de Manises, 46940 Valencia, Spain; nmaroto@hospitalmanises.es; 69 Department of Gastroenterology, Hospital Royo Villanova, 50015 Zaragoza, Spain; jmnerindlp@gmail.com; 70 Department of Gastroenterology, Complexo Hospitalario Universitario Xeral-Calde de Lugo, 27004 Lugo, Spain; elecortiz@yahoo.es; 71 Department of Gastroenterology, Hospital General Universitario Gregorio Marañón, Instituto de Investigación Biomédica Gregorio Marañón (IiSGM), 28007 Madrid, Spain; drnachomarin@hotmail.com; 72 Department of Gastroenterology, Hospital Universitario Infanta Cristina, 28981 Madrid, Spain; belen.botella@salud.madrid.org; 73 Department of Gastroenterology, Hospital de Manacor, 07500 Manacor, Spain; asapina@hospitalmanacor.org; 74 Department of Gastroenterology, Hospital Doctor José Molina Orosa, 35500 Lanzarote, Spain; noelia_ingenio@hotmail.com; 75 Department of Gastroenterology, Hospital Comarcal Sierrallana, 39300 Torrelavega, Spain; jlforce2@yahoo.es; 76 Department of Gastroenterology, Hospital Santa Bárbara, 13500 Puertollano, Spain; bumidi@hotmail.com; 77 Department of Gastroenterology, Hospital Rey Juan Carlos, 28933 Madrid, Spain; castamilla@gmail.com; 78 Department of Gastroenterology, Hospital Universitario de Getafe, 28905 Madrid, Spain; veronica.opio@salud.madrid.org; 79 Department of Gastroenterology, Hospital General Universitario Reina Sofía, 30003 Murcia, Spain; isabelndp@hotmail.com; 80 Department of Gastroenterology, Hospital Reina Sofía, 31500 Tudela, Spain; marcos.kutz.leoz@navarra.es; 81 Department of Gastroenterology, Hospital Puerta del Mar, 11009 Cádiz, Spain; aljabbe@gmail.com; 82 Department of Gastroenterology, Hospital de la Santa Creu i Sant Pau, 08041 Barcelona, Spain; jgordillo@santpau.cat; 83 Department of Gastroenterology, Hospital San Jorge, 22004 Huesca, Spain; ybernieto@gmail.com; 84 Department of Gastroenterology, Hospital San Juan de Dios del Aljarafe, 41930 Sevilla, Spain; ytgastrosur@gmail.com; 85 Department of Gastroenterology, Hospital Can Misses, 07800 Ibiza, Spain; mnovella@asef.es; 86 Department of Gastroenterology, Hospital Sant Joan de Deu, 08950 Barcelona, Spain; silvia36455@live.com; 87 Department of Gastroenterology, Hospital Universitari Sant Joan, 25001 Lérida, Spain; fjmartinez@grupsagessa.com; 88 Department of Gastroenterology, Hospital General de La Palma, 38713 Santa Cruz de Tenerife, Spain; lkolle@hotmail.com; 89 Department of Gastroenterology, Hospital Santa Caterina, 17190 Gerona, Spain; miriam.sabat@ias.cat; 90 Department of Gastroenterology, Hospital Palamós, 17230 Girona, Spain; Cesar_kike@hotmail.com; 91 Department of Gastroenterology, Hospital Comarcal de Inca, 07300 Inca, Spain; eduardoy.iyo@hcin.es; 92 Department of Gastroenterology, Hospital General La Mancha Centro, 13600 Ciudad Real, Spain; dr.roncero@gmail.com; 93 Department of Gastroenterology, Hospital García Orcoyen, 31200 Estella, Spain; rebeca.irisarri.garde@navarra.es; 94 Department of Gastroenterology, Hospital Sagrat Cor, 08029 Barcelona, Spain; laia.lluis@quironsalud.es; 95 Department of Gastroenterology, Hospital de Torrejón, 28850 Madrid, Spain; isa.blazquezgomez@gmail.com; 96 Department of Gastroenterology, Hospital de Mendaro, 20850 Guipuzkoa, Spain; evazapatamorcillo@gmail.com; 97 Department of Gastroenterology, Hospital Obispo Polanco, 44002 Teruel, Spain; mjaescriche@gmail.com; 98 Department of Gastroenterology, Hospital General Universitario Los Arcos del Mar Menor, San Javier, 30739 Murcia, Spain; cristina.martinezpascual@gmail.com; 99 Department of Gastroenterology, Hospital General de Villarobledo, 02600 Albacete, Spain; montealegre_m@hotmail.com; 100 Department of Gastroenterology, Hospital Medina del Campo, 47400 Valladolid, Spain; lauramataroman@gmail.com; 101 Department of Gastroenterology, Hospital de Montilla, 14550 Córdoba, Spain; ammonrobel@ephag.es; 102 Department of Gastroenterology, Hospital Quirón Costa Adeje, 38660 Santa Cruz de Tenerife, Spain; Dr.alejandrohc@gmail.com; 103 Department of Gastroenterology, Hospital Santos Reyes, 09400 Aranda de Duero, Spain; luishernandezvillalba@gmail.com; 104 Department of Gastroenterology, Clínica Astarté, 11011 Cádiz, Spain; maria.tejada.cabrera@gmail.com; 105 Department of Gastroenterology, Hospital Ernest Lluch, 50299 Zaragoza, Spain; albertomirsubias@gmail.com; 106 Department of Gastroenterology, Hospital Central de La Cruz Roja San José y Santa Adela, 28003 Madrid, Spain; marisagalve@gmail.com; 107 Department of Gastroenterology, Hospital San Juan De Dios, 38009 Tenerife, Spain; msolerrguez@yahoo.es; 108 Department of Gastroenterology, Hospital Virgen de Altagracia, 13002 Manzanares, Spain; danielhervias@gmail.com; 109 Department of Gastroenterology, Hospital Dexeus, 08028 Barcelona, Spain; jagomez@idexeus.es; 110 Department of Preventive Medicine and Public Health, School of Medicine, Universidad Autónoma de Madrid/IdiPaz and CIBERESP, 28029 Madrid, Spain; fernando.artalejo@uam.es (F.R.-A.); esthergge@gmail.com (E.G.-E.)

**Keywords:** epidemiology, incidence, inflammatory bowel disease, Crohn’s disease, ulcerative colitis

## Abstract

(1) Aims: To assess the incidence of inflammatory bowel disease (IBD) in Spain, to describe the main epidemiological and clinical characteristics at diagnosis and the evolution of the disease, and to explore the use of drug treatments. (2) Methods: Prospective, population-based nationwide registry. Adult patients diagnosed with IBD—Crohn’s disease (CD), ulcerative colitis (UC) or IBD unclassified (IBD-U)—during 2017 in Spain were included and were followed-up for 1 year. (3) Results: We identified 3611 incident cases of IBD diagnosed during 2017 in 108 hospitals covering over 22 million inhabitants. The overall incidence (cases/100,000 person-years) was 16 for IBD, 7.5 for CD, 8 for UC, and 0.5 for IBD-U; 53% of patients were male and median age was 43 years (interquartile range = 31–56 years). During a median 12-month follow-up, 34% of patients were treated with systemic steroids, 25% with immunomodulators, 15% with biologics and 5.6% underwent surgery. The percentage of patients under these treatments was significantly higher in CD than UC and IBD-U. Use of systemic steroids and biologics was significantly higher in hospitals with high resources. In total, 28% of patients were hospitalized (35% CD and 22% UC patients, *p* < 0.01). (4) Conclusion: The incidence of IBD in Spain is rather high and similar to that reported in Northern Europe. IBD patients require substantial therapeutic resources, which are greater in CD and in hospitals with high resources, and much higher than previously reported. One third of patients are hospitalized in the first year after diagnosis and a relevant proportion undergo surgery.

## 1. Introduction

Inflammatory bowel diseases (IBD)—Crohn’s disease (CD), ulcerative colitis (UC) and inflammatory bowel disease unclassified (IBD-U)—are chronic inflammatory conditions that mainly affect the gastrointestinal tract but might also involve other organs, so they are considered systemic diseases. IBD is mainly diagnosed in young people, and is associated with significant morbidity and disability [1].

In the last decades, the global burden of IBD has increased due to its growth in newly industrialized countries, increased rates of diagnosis and decline in mortality [1]. In a recent systematic review, authors found that the prevalence of IBD today exceeds 0.3% of the total population in countries like Canada, the United States, New Zealand, Denmark, Germany and the United Kingdom [2]. Furthermore, a predictive model for Canada revealed that the prevalence of IBD in 2025 could rise to 0.9% [3]. The growing disease burden is aggravated by increasing costs in the last years. In recent years, the therapeutic target in IBD management has moved from control of symptoms to mucosal healing [4] and more effective therapeutic, but also costly, options have been introduced [5,6]. For this reason, new epidemiological evidence is needed to better understand the penetration of new therapeutic algorithms.

Nationwide epidemiological studies are necessary to ensure representativeness and limit the impact of regional variability on the results. However, there is a paucity of nationwide epidemiological studies on IBD; in Europe, most of them have been carried out in countries with relatively small populations in Northern or Central Europe [7,8,9], and have seldom included data on Southern Europe countries. In addition, most nationwide studies are based on administrative data being at risk of exposure to misclassification or confounding bias [10].

In Europe, the EpiCom/Epi-IBD study was initiated in 2010 to investigate the differences in epidemiology and management of IBD between Eastern and Western countries. A total of 1515 IBD patients were included over 2010, reporting an incidence of 15 cases/100,000 person-years [11]. In Spain, only one center was involved in the EpiCom study, and previous epidemiological studies were performed many years ago, in selected areas, and included a small number of patients [12].

The aims of the present study were to assess the incidence of IBD in Spain, describe the characteristics of patients at diagnosis and their use of immunosuppressive treatments and biological drugs, and measure the cumulative incidence of surgeries and hospital admissions during the first year after IBD diagnosis. 

## 2. Methods

### 2.1. Study Design

EpidemIBD (see Appendix A) is a prospective, population-based incidence cohort of adult patients aiming to assess the incidence and clinical characteristics of IBD (CD, UC, or IBD-U), in Spain. In addition, each incident case is being followed to determine changes in disease phenotype or location, the exposure to immunosuppressive and biologic treatments, and the need for hospital admission or surgery during the first 5 years after diagnosis. In the present study, we describe baseline characteristics of study participants, and provide data on the cumulative incidence of treatment use, hospitalizations and surgeries during the first 12 months after diagnosis. The study protocol, which has already been published [12], was approved by the Research Ethics Committee of the coordinating hospital (Hospital Universitario de La Princesa, Madrid). All patients provided written informed consent to participate.

### 2.2. Study Population

As previously described in the protocol, adult (≥18 years of age) IBD patients diagnosed between 1 January 2017 and 31 December 2017 in the study centers were included [12]. Diagnosis of IBD was based on European Crohn’s and Colitis Organisation criteria [13,14]. Patients were eligible to be included in the IBD incident cohort if they belonged to the reference area of the participating centers. A patient diagnosed at one center (e.g., a particular hospital in Madrid) who was part of the reference population of another center (e.g., a different hospital in Madrid) was assigned to his/her own reference population (in this example, that of his/her usual hospital).

### 2.3. Recruitment and Patient Follow-Up

This study is being conducted at 108 centers, 100 of them within the National Health System (providing free access to healthcare). The initial selection of centers was made from the database of health centers of the Ministry of Health, which included 893 centers in 2016. A total of 205 centers were selected to start the study. Centers that did not adequately follow the study procedures or ensure the inclusion of all patients diagnosed with IBD in their area were excluded [12]. Finally, 108 centers continue to be involved in the study, covering a referral area with a population of 22,270,357 inhabitants (approximately 50% of Spanish population, which was 46,659,302 as of 28 June 2018) [15]. Once an incident patient was included in the registry, two additional visits, at 3 and 12 months, were performed during the first year to confirm the diagnosis and to update the information regarding disease extent and behavior, medical treatments, hospitalizations and surgeries. Patients are being followed-up every 6 months until completing 5 years from IBD diagnosis. Misdiagnosed patients were excluded from the analysis. Remote monitoring was performed by the research staff to ensure data quality.

### 2.4. Definitions

IBD location and phenotype were defined according to the Montreal classification [16]. Time to diagnosis was defined as the time from the first medical consultation by a symptomatic patient to the diagnosis of IBD.

Socioeconomic level was assessed considering the patient’s educational level (primary or lower, secondary, higher education), occupational (self-employed, employed, unemployed, retired) and professional status (non-salaried or salaried). The number of cohabitants at the patient’s home during his/her childhood (up to 16 years) and at diagnosis of IBD was also recorded.

Smoking status was categorized at the time of diagnosis of IBD as “nonsmoker”, “former smoker”, or “smoker”. Patients were considered “smokers” if they smoked more than 7 cigarettes per week for at least 6 months and had smoked at least 1 cigarette in the 6 months prior to diagnosis. Patients were considered “ex-smokers” if they quit smoking at least 6 months before diagnosis. Patients were considered “nonsmokers” if they never smoked or did so in very small amounts or occasionally [17].

Hospitals were clustered in conglomerates taking into account different variables provided in the database from the Spanish Ministry of Health, such as provision, offer of services, activity, complexity or teaching, which established the following five categories: (1) Small general hospitals (less than 150 beds on average, hardly any high-tech resources, low complexity); (2) Medium general hospitals (average size less than 200 beds, minimal technological resources, some teaching activity and more complexity); (3) General hospitals (of average size around 500 beds, medium complexity); (4) Referral hospitals (large hospitals but heterogeneous in resources, size and activity, great teaching activity and high complexity); and (5) Large referral hospitals (great structural weight and a lot of activity, full offer of services, more than 600 doctors and around 300 residents). To analyze the impact of the hospital category on patients’ care, patients were considered belonging to the center that included them in the registry, in case it was different from the patients’ referral center.

### 2.5. Data Collection and Follow-Up

Study data were collected and managed using an electronic data capture tool (Research Electronic Data Capture [REDCap]) [18], which is hosted at Asociación Española de Gastroenterología (AEG; www.aegastro.es, accessed date 15 May 2021), a non-profit medical society. AEG provided this service free of charge, with the aim of promoting investigator-driven research.

### 2.6. Statistical Analysis

The reference population (based on estimates form the National Statistics Office) for the analyses is the population of the catchment areas of the participating centers [15]. The incidence rate (number of incident cases per 100,000 inhabitants) during one year was calculated using the reference population as denominator.

Mean and standard deviation or median and interquartile range (IQR) were calculated for quantitative variables, depending on whether they were normally distributed or not. Categorical variables were compared using Chi-square (χ^2^) test and quantitative variables using the appropriate test. Qualitative variables were compared using the chi-square (χ^2^) test and the Fisher’s exact test.

The Kaplan–Meier method was used to estimate the time course of treatment use, hospital admissions and surgery; the differences between the curves were assessed with the log-rank test. Use of different therapeutic choices was compared based on the type of IBD and hospital characteristics (see above). The main aim of this study was to calculate the incidence of IBD in Spain and to describe the characteristics and the use of treatments, surgeries and hospitalisations in this population; therefore, no multivariate analysis was performed.

## 3. Results

### 3.1. Incidence of IBD

**Figure 1:**
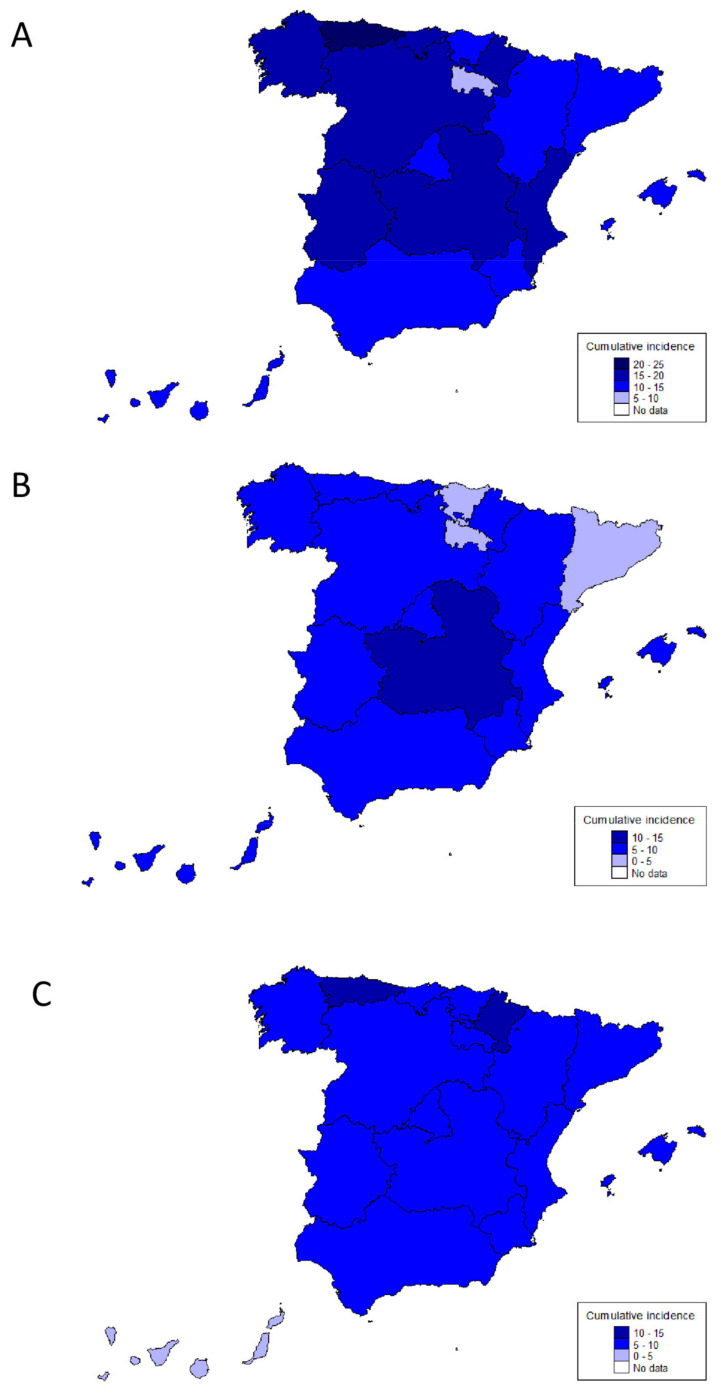
Incidence of inflammatory bowel disease (**A**), Crohn’s disease (**B**) and ulcerative colitis (**C**) by Autonomous Communities in Spain in 2017 (cases/100,000 person-years).

A total of 3611 incident cases of IBD diagnosed during 2017 in 108 hospitals covering over 22 million adult inhabitants (about 50% of the Spanish population) comprise the study cohort (Table 1). The overall cumulative incidence (cases/100,000 person-years) during the first year of follow-up was 16.2 for IBD, 7.4 for CD, 8.1 for UC, and 0.7 for IBD-U (Figure 1). Incidence of CD was somewhat higher in Central Spain, while that of UC was higher in Northern Spain (Asturias and Navarra) (Figure 1 and Supplementary Table S1).

### 3.2. Patients’ Characteristics

Table 1 shows the main baseline characteristics of participants. Approximately 50% of them were male, with a median age of 42 years. A total of 1807 (50%) patients had UC, 1647 (46%) had CD, and 156 (4%) IBD-U. Around 8% of patients were asymptomatic at diagnosis. Median diagnosis delay (from symptom onset to IBD diagnosis) was 3 months (IQR = 1–9 months). Median time from primary care consultation to IBD diagnosis was 2 months (IQR = 1–6 months), and from gastroenterologist consultation to IBD diagnosis 0 months (IQR = 0–1 month). Family history of IBD was present in 525 patients (15%): 441 (12%) had one and 84 (2.5%) more than one relative with IBD. In total, 175 (4.8%) patients had first-degree relatives with IBD. In 176 (4.9%) patients, the closest relatives with IBD were second-degree relatives, and in 173 (4.8%) third-degree relatives. A total of 327 (9%) patients had extraintestinal manifestations at diagnosis. The most frequent was peripheral arthropathy (4%), followed by skin manifestations (1.8%), and spondiloarthropathy (1.3%) (Supplementary Table S2).

In patients with CD, the majority of patients had ileal (55%) or ileocolonic (26%) involvement (Table 1). Inflammatory behavior was the most prevalent in our cohort (82%); however, 11% had stricturing and 7% fistulizing behavior already at IBD diagnosis. With respect to UC patients, 31% had extensive colitis and 31% left-sided colitis. Examinations performed to diagnose IBD are shown in Supplementary Table S3. Almost all patients (99%) underwent colonoscopy, 17% CT-scan, 15% magnetic resonance enterography, 7.3% upper gastrointestinal endoscopy and 4.5% abdominal ultrasound.

The diagnosis delay was longer for CD than for UC (5 vs. 2 months, *p* < 0.01; Table 2). The proportion of patients with symptoms at diagnosis was higher in UC than in CD (94 vs. 89%, *p* < 0.01). By contrast, those with CD had higher frequency of family history of the disease that those with UC (18 vs. 13%, *p* < 0.01), former smokers (38 vs. 12%, *p* < 0.01) and extraintestinal manifestations (12 vs. 6%, *p* < 0.01).

### 3.3. Drug Treatment and Surgery during the First 12 Months after Diagnosis

**Figure 2:**
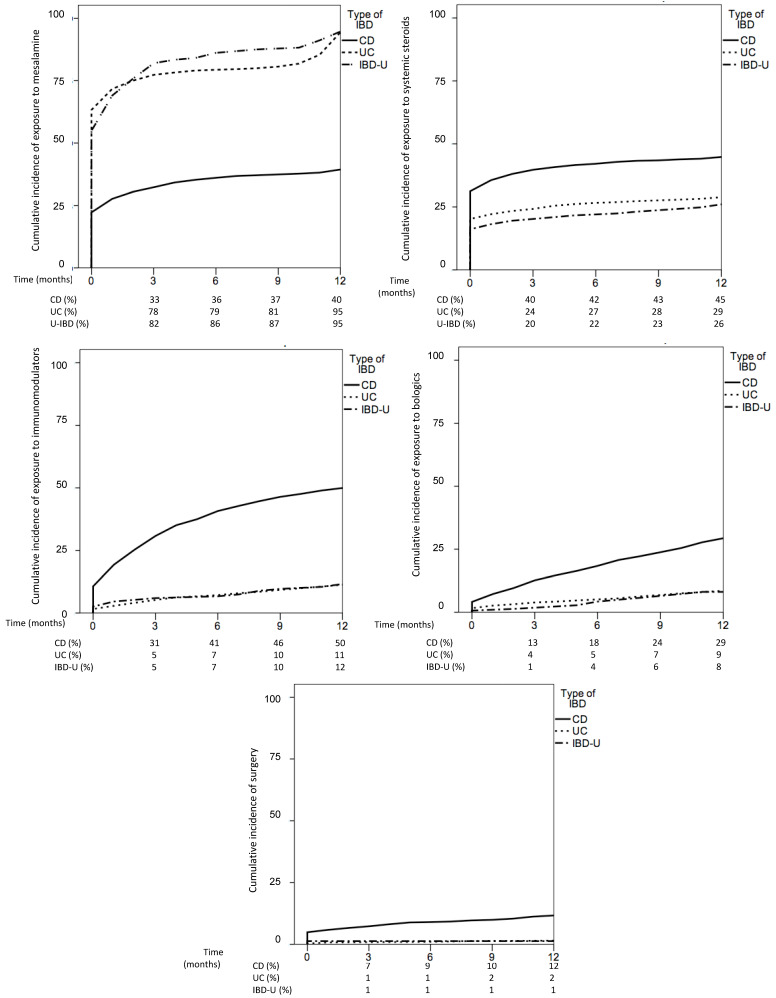
Cumulative incidence of exposure to mesalamine (**1**), systemic steroids (**2**), immunomodulators (**3**), biologics (**4**) and surgery (**5**) in Crohn’s disease (CD), ulcerative colitis (UC) and inflammatory bowel disease unclassified (IBD-U) during 1-year follow-up.

About 35% of patients had received systemic steroids, 26% immunomodulators and 15% biological drugs (Table 3). When comparing CD and UC, while the cumulative incidence of exposure to mesalamine was significantly higher in UC and IBD-U, the use of steroids, immunomodulators and biologics was significantly higher in CD (Figure 2).

A total of 199 (5%) patients underwent surgery; 170 patients (4.7%) were operated once, 20 (0.6%) twice, 7 (0.2%) three times, and 2 (0.1%) four times within the first 12 months. The cumulative incidence of surgery was higher in CD than UC (11 vs. 1.3%, *p* < 0.01) (Figure 2).

### 3.4. Hospitalizations

A total of 1012 (28%) patients were hospitalized within the first 12 months: 585 (35%) in CD and 391 (21%) in UC. The main reasons for hospital admission are summarized in Supplementary Table S4. Eight-hundred fifteen (23%) patients were hospitalized once, 151 (4.2%) twice, 33 (0.9%) three times and 13 (0.5%) four times.

### 3.5. Drug Treatments, Surgery and Hospitalizations during the First 12 Months Based on Hospital Category

Of the 108 participating hospitals, 100 were publicly funded by the National Health System and 8 of them were privately funded. Two (2%) hospitals were classified into category 1 (lowest resources), 4 (4%) into category 2, 44 (40%) into category 3, 31 (29%) into category 4, and 27 (25%) into category 5 (highest resources).

**Figure 3:**
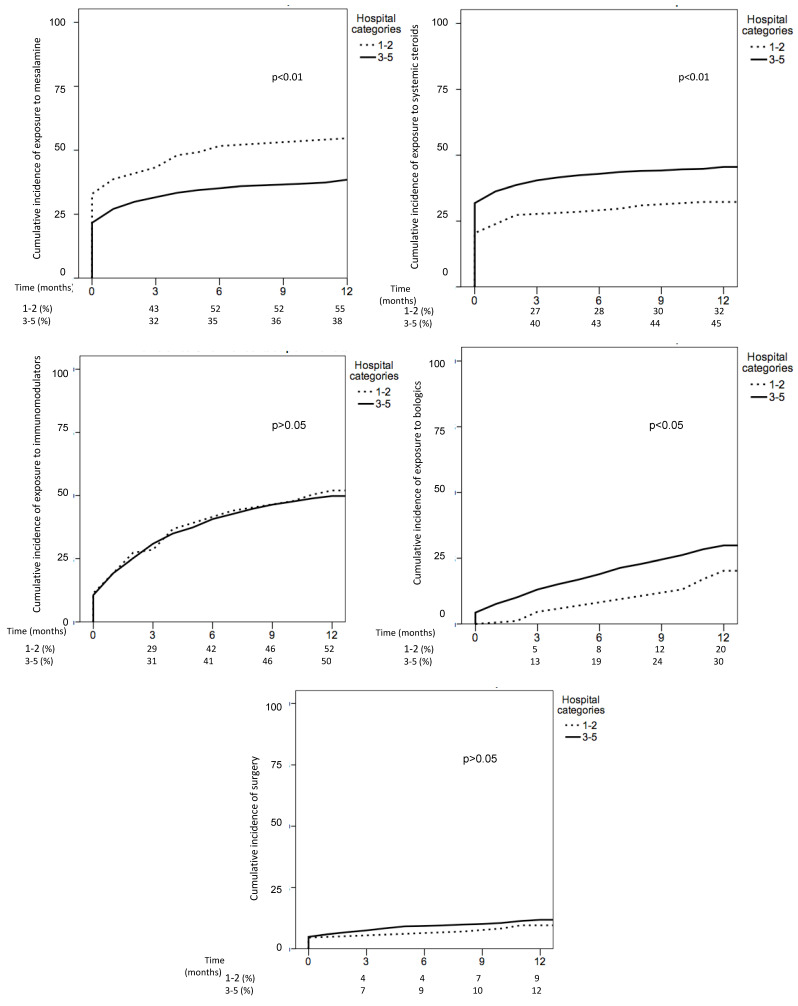
Cumulative incidence of treatments in Crohn’s disease (CD) based on hospital categories: lower resources (**1** and **2**) vs. higher resources (**3**–**5**).

**Figure 4:**
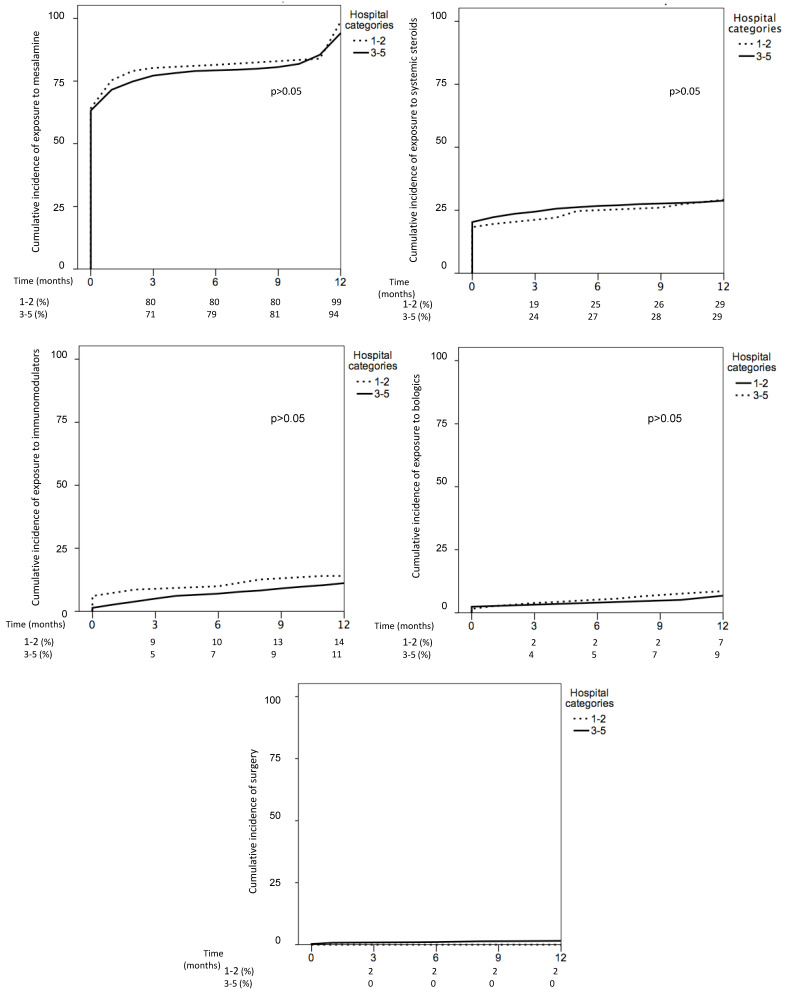
Cumulative incidence of treatments in ulcerative colitis based on hospital categories: low resources (**1** and **2**) vs. higher resources (**3**–**5**).

In total, 177 patients were treated in hospitals with low resources (categories 1–2) and 3434 patients in hospitals with high resources (categories 3–5). The main characteristics of IBD patients were similar in both types of hospitals (Table 4). With respect to CD management, use of some drug treatments was different between hospitals with low and high resources (Table 5). Thus, the cumulative incidence of exposure to mesalamine in CD was higher in hospitals with low resources, while the cumulative incidence of exposure to systemic steroids was higher in hospitals with high resources (mainly due to higher proportion of patients starting systemic steroids at IBD diagnosis). In addition, use of biological drugs was also higher in CD patients from hospitals with high resources (Figure 3). The cumulative incidence of surgery and hospitalizations was similar in both groups. Regarding UC, the cumulative incidence of treatment with mesalamine, steroids, immunomodulators, biologics and surgery was similar in hospitals with low and high resources (Figure 4).

## 4. Discussion

In this population-based study we evaluated the incidence, clinical characteristics and treatment choices in newly diagnosed IBD patients. To our knowledge, this is one of the largest studies on IBD epidemiology, and provides a comprehensive analysis of the current characteristics, treatments, surgery and hospitalization in a Southern European country. This study will be a helpful resource for researches and caregivers to understand the epidemiology, the clinical characteristics and the treatments choices in the biologics era.

Our main finding was that the incidence of IBD in Spain was 16.2 cases per 100,000 inhabitant-years, considerably higher than previously described in Spain and even higher that previously reported in Western European countries [2,19]. For instance, in the 2011 inception cohort of the EpiCom study, the incidence of IBD was 7.2 cases/100,000 person-years in Greece, 10.5 in Italy, and 11.8 in Portugal.

We have confirmed the results of previous studies, showing that the median diagnosis delay was 3 months and was significantly higher for CD than for UC [20,21]. In addition, we have been able to assess whether the delay depends on the time from the symptoms onset to the consultation with the primary care physician or on the access to the gastroenterologist. When we split the overall diagnosis delay into patient-dependent and physician-dependent intervals, we found that the first one was longer. In a study performed by Cantoro et al. in an Italian population, authors found similar results: time from symptoms onset to first consultation was about 3 times longer than time from first consultation to diagnosis. Authors suggested that the high prevalence of irritable bowel syndrome-like symptoms at IBD onset may be responsible for the underestimation of IBD symptoms by patients, leading to longer time to first consultation, while higher awareness of the disease once the patient consults with the physician might help to shorten delayed diagnosis [20].

Another major finding in our study is the high proportion of exposure of our population to immunomodulators and biological agents, much higher than previously reported. This finding is of great importance due to the high costs associated with IBD management. Several studies have observed a marked increase in the use of immunosuppressive and biological drugs in previous decades [22]. For instance, in the EpiCom cohort, 12% of patients with CD in Western Europe received immunomodulators at 12 months after diagnosis, and 66% at 5 years. Regarding biological agents, 19% of CD patients received biological drugs at 12 months and 33% at 5 years after diagnosis in the EpiCom cohort. In the case of UC, 5 years after the diagnosis, 29% had been exposed to immunomodulators and 11% to biological drugs.

In contrast, in our cohort, over 25% of patients received immunomodulators and over 15% biological drugs in the first year after IBD diagnosis. Furthermore, these figures were higher in CD than in UC. In this sense, one year after diagnosis, as much as 50% of CD patients and 11% of UC patients had used immunomodulators. Similarly, 29% of CD and 8% of UC patients were treated with biological drugs. Newer therapeutic algorithms that aim to achieve mucosal healing instead of only symptomatic control are probably increasing the use of biological therapies among patients [23]. Our results might represent more accurately the current use of IBD therapeutic choices by unselected gastroenterologists.

Two recent publications have shown that IBD-related healthcare costs, in both Europe and North America, are mainly attributable to medical costs, primarily biological drugs [5,6]. In the EpiCom cohort, with lower use of biologic than in our cohort, authors found an increase in expenditure on biologics during the 5-year follow-up. Biologics costs accounted for 73% of costs in CD and 48% in UC, with a mean cost per patient-year for biologics of 866 euros [6]. In Canada, in the decade between 2005 and 2015, the annual costs of care ascribed to IBD management nearly tripled (from $14.1 M to $38.7 M). Most of this increase was attributable to the rising costs of anti-TNF drug prescription. For an adequate allocation of resources, policy makers and managers in the context of a universal publicly funded health system should be aware of IBD burden when planning the costs of care for these patients.

To the best of our knowledge, our study is the first showing treatment differences in patients with CD depending on hospital complexity. Use of biological drugs was higher in hospitals with high resources than in those with lower resources. It is unlikely that this is due to a selection bias of the most complex patients in hospitals with higher resources, since the patients have been treated in the same hospital since the disease was diagnosed, when the clinical evolution was not fully predictable yet. Rather, it may be due to greater “aggressiveness” of treatment to control the disease in these centers, as is also reflected in more frequent use of systemic corticosteroids and less use of mesalamine in patients with CD.

Biological drugs seem to be associated, at least in the short term, with a lower need for surgery and hospital admissions [24]. However, long-term results, in real-life populations, are more controversial. Some studies have observed a decrease in the need for hospital admissions and surgery associated with an increase in the use of biological drugs, while other studies have not confirmed these differences [5,22,25,26,27,28]. In our study, a third of the patients required hospital admission during the first year from diagnosis, and 5.5% underwent at least one surgical intervention (11% in CD). Our results are in line with other previously reported results [25,26]. Despite higher use of biologics in hospitals with higher resources, we found no differences in the risk of surgery in comparison with centers with lower resources. However, the results of our study correspond to the first year since diagnosis; follow-up of the EpidemIBD cohort is ongoing until completing 5 years from diagnosis, which will allow us to know if early treatment with immunosuppressive and biological drugs is associated with a change in the natural history of the disease.

Our study has several limitations. The number of centers located in rural areas and the number of hospitals with less resources was low; however, due to the large number of participating centers we could compare the incidence of IBD and the management of IBD in different types of hospitals. In addition, the registry lacks of data on clinical and endoscopic severity; however, to assess the appropriateness of immunomodulators and biologics use was not an aim of our study. At the time of the present analysis, patient follow-up was limited to one year and it was not possible to assess whether the use of immunomodulators and biologics had an impact on the natural history of the disease. The EpidemIBD registry is ongoing, as patient follow-up will last 5 years. Therefore, we will be able to assess the impact of biological and immunosuppressive treatments on the evolution of the disease. The main strengths of the present study are the large number of participating centers (108 hospitals) and patients (3611), and the prospective inclusion and follow-up of patients diagnosed within well-defined administrative areas covering over 20 million adult inhabitants. Patients were followed-up in a standardized manner and were comparable in observation time. In addition, the full database was monitored remotely to resolve queries and ensure data quality. Finally, our study included unselected centers (not only reference hospitals with a high level of patient complexity but also regional hospitals), which provides an accurate representation of current IBD management.

In conclusion, in this prospective population-based cohort, the incidence of IBD in a Southern European country is over 16 cases/100,000 person-years, higher than previously described in Spain and in other Western European countries. The use of biological drugs is much higher than previously reported, mainly in CD patients and in hospitals with high resources. The EpidemIBD study underscores the importance of IBD in healthcare systems, which have to manage this complex disease.

## Figures and Tables

**Table 1:** Characteristics of the inception cohort.

	Overall *n* = 3611
Age, years (median, IQR)	42 (30–55)
Male gender, *n* (%)	1908 (53)
Former smokers, *n* (%)	880 (24.5)
Symptoms at diagnosis, *n* (%)	3280 (92)
Diagnostic delay, months (median, IQR)	3 (1–9)
Family history of IBD, *n* (%)	524 (15)
Educational level	
Primary or none	1220 (31)
Secondary	1424 (41)
University degree	961 (28)
Employment status	
Self-employed	351 (10)
Employee	1755 (51)
Unemployed	532 (15.4)
Others	326 (9.5)
Extraintestinal manifestations, *n* (%)	327 (9)
Crohn’s disease, *n* (%)	1647 (46)
Ileal, *n* (%)	900 (55)
Colonic, *n* (%)	312 (19)
Ileocolonic, *n* (%)	431 (26)
Upper gastrointestinal tract, *n* (%)	52 (3)
Inflammatory, *n* (%)	1347 (82)
Stricturing, *n* (%)	183 (11)
Fistulizing, *n* (%)	114 (7)
Perianal, *n* (%)	185 (11)
Ulcerative colitis, *n* (%)	1807 (50)
Extensive, *n* (%)	563 (31)
Left-sided colitis, *n* (%)	563 (31)
Proctitis, *n* (%)	678 (38)
Unclassified inflammatory bowel disease, *n* (%)	156 (4)

Interquartile range, IQR; inflammatory bowel disease, IBD.

**Table 2:** Characteristics of the inception cohort based on inflammatory bowel disease type (Crohn’s disease vs. ulcerative colitis).

	Crohn’s Disease *n* = 1647	Ulcerative Colitis *n* = 1807	*p*
Age, years (median, IQR)	41 (28–54)	46 (34–57)	<0.05
Female gender, *n* (%)	821 (50)	808 (45)	<0.05
Former smokers, *n* (%)	630 (38)	217 (12)	<0.05
Symptoms at diagnosis, *n* (%)	1465 (89.5)	1675 (94)	<0.05
Diagnostic delay, months (median, IQR)	5 (1–15)	2 (1–5)	<0.05
Family history of IBD, *n* (%)	288 (18)	225 (13)	<0.05
Extraintestinal manifestations, *n* (%)	204 (12)	114 (6)	<0.05

Interquartile range, IQR; inflammatory bowel disease, IBD.

**Table 3:** Use of inflammatory bowel disease drugs, surgery and hospitalizations in the entire cohort (**A**) and based on inflammatory bowel disease type (**B**) in the first year after diagnosis.

(A)			
Total Number of Patients	*n* = 3611		
Mesalamine, *n* (%)	2450 (68)		
Steroids, *n* (%)	1916 (53)		
Systemic steroid therapy, *n* (%)	1252 (35)		
Immunomodulators, *n* (%)	936 (26)		
Thiopurines, *n* (%)	860 (24)		
Methotrexate, *n* (%)	114 (3.2)		
Cyclosporine, *n* (%)	13 (0.4)		
Tofacitinib, *n* (%)	2 (0.1)		
Biologics, *n* (%)	558 (15.5)		
Anti-TNF, *n* (%)	535 (14.8)		
Ustekinumab, *n* (%)	24 (0.7)		
Vedolizumab, *n* (%)	34 (0.9)		
Surgery, *n* (%)	199 (5.5)		
Hospital admissions, *n* (%)	1012 (28)		
**(B)**			
	**Crohn’s Disease *n* = 1647**	**Ulcerative Colitis *n* = 1807**	***p***
Mesalamine ever, *n* (%)	625 (38)	1681 (73)	<0.01
Steroids ever, *n* (%)	1170 (71)	688 (38)	<0.01
Systemic steroid therapy, *n* (%)	717 (43.5)	497 (27.5)	<0.01
Immunomodulators, *n* (%)	746 (45)	174 (10)	<0.01
Biologics, *n* (%)	415 (25)	132 (7)	<0.01
Surgery, *n* (%)	174 (11)	23 (1.3)	<0.01
Hospital admissions, *n* (%)	585 (35.5)	391 (22)	<0.01

**Table 4:** Patients’ characteristics based on hospital categories.

	Low Resources (Categories 1–2) *n* = 177	High Resources (Categories 3–5) *n* = 3434	*p*
Age, years (median, IQR)	45 (31–55)	43 (31–56)	>0.05
Male gender, *n* (%)	101 (57)	1807 (53)	>0.05
Never smokers, *n* (%)	56 (32)	1410 (41)	0.01
Symptoms at diagnosis, *n* (%)	166 (94.3)	3114 (92)	>0.05
Diagnostic delay, months (median, IQR)	4 (1–15)	3 (1–8)	>0.05
Time from symptoms onset to primary care consultation, months (median, IQR)	2 (0–6)	2 (1–6)	>0.05
Time from primary care to gastroenterologist consultation, months (median, IQR)	1.5 (0–3)	2 (0–5)	>0.05
Family history of IBD, *n* (%)	24 (14)	501 (15)	>0.05
Extraintestinal manifestations, *n* (%)	23 (13)	304 (9)	>0.05
Crohn’s disease, *n* (%)	88 (50)	1559 (45.5)	>0.05
Ileal, *n* (%)	57 (65)	843 (54)	>0.05
Colonic, *n* (%)	10 (11)	302 (19.5)	>0.05
Ileocolonic, *n* (%)	21 (24)	410 (26)	>0.05
Upper gastrointestinal tract, *n* (%)	1 (1)	51 (3)	>0.05
Inflammatory, *n* (%)	76 (86)	1271 (82)	>0.05
Stricturing, *n* (%)	9 (10)	174 (11)	
Fistulizing, *n* (%)	3 (4)	111 (7)	
Perianal, *n* (%)	4 (4.5)	181 (11.7)	0.04
Ulcerative colitis, *n* (%)	82 (46)	1725 (50)	>0.05
Extensive, *n* (%)	25 (31)	538 (31)	
Left-sided colitis, *n* (%)	24 (29)	539 (31)	>0.05
Proctitis, *n* (%)	33 (40)	645 (38)	
Unclassified inflammatory bowel disease, *n* (%)	7 (4)	149 (4.5)	>0.05

Interquartile range, IQR; inflammatory bowel disease, IBD.

**Table 5:** Prevalence of exposure to different drug treatments, surgery and hospitalizations based on hospital categories.

	Low Resources (Categories 1–2) *n* = 177	High Resources (Categories 3–5) *n* = 3434	*p*
Mesalamine ever, *n* (%)	136 (77)	2314 (67.4)	<0.01
Steroids ever, *n* (%)	102 (58)	1814 (53)	>0.05
Systemic steroid therapy, *n* (%)	53 (30)	1199 (35)	>0.05
Immunomodulators, *n* (%)	56 (32)	880 (26)	>0.05
Biologics, *n* (%)	22 (12)	536 (16)	>0.05
Surgery, *n* (%)	8 (4.5)	191 (5.6)	>0.05
Hospital admissions, *n* (%)	39 (22)	973 (28)	>0.05

## Data Availability

The data underlying this article cannot be shared publicly due to the privacy of individuals that participated in the study. The data will be shared on reasonable request to the corresponding author.

## Supplementary Materials

The following are available online at https://www.mdpi.com/2077-0383/10/13/2885/s1, Supplementary Table S1. Incidence rates (cases/100,000 person-years) for inflammatory bowel disease by Autonomous Community in Spain, 2017; Supplementary Table S2. Extraintestinal manifestations at diagnosis in the inception cohort; Supplementary Table S3. Examinations performed at inflammatory bowel disease diagnosis; Supplementary Table S4. Reasons for hospitalizations in the first year after diagnosis; Supplementary Table S5. Patients’ characteristics based on hospital categories.

## Abbreviations

Inflammatory bowel disease, IBD; Crohn’s disease, CD; ulcerative colitis, UC; inflammatory bowel disease unclassified, IBD-U; interquartile range, IQR.

## Appendix A

**The EpidemIBD Study Group Of Geteccu:**

Alfredo Abraldes Bechiarelli, José Manuel Benítez, María del Rosario Calderón, Raquel Camargo, Álvaro Hernández-Martínez, Eva Iglesias Flores, Eduardo Leo Carnerero, Sandra Marín Pedrosa, Ana Monrobel, Andrea Núñez Ortiz, Natalia Ruiz Santana, María Tejada, Yolanda Torres Domínguez, María José Alcalá, Erika Alfambra, Yolanda Ber, Fernando Gomollón, Miguel Montoro, Juan Manuel Nerín, Elena Peña, Raquel Vicente, José María Duque, Ruth de Francisco, Alejo Mancebo Mata, Susana Martínez González, Isabel Pérez Martínez, Sabino Riestra, Pilar Varela Trastoy, Inmaculada Alonso-Abreu, Daniel Ceballos, Noelia Cruz, Elena Guerra del Río, Alejandro Hernández Camba, Lilian Kole, José Miguel Marrero, Nuria Pérez, Marta Soler, Milagros Vela, José Luis F. Forcelledo, Montserrat Rivero, Rufo Lorente Poyatos, Cristina Verdejo Gil, María Montealegre, Alfredo J. Lucendo, Óscar Roncero, Abdel Bouhmidi, Daniel Hervías, Lara Arias García, Jesús Barrio, Ana Y. Carbajo, Luis Fernández-Salazar, Paola Fradejas, Ana María Fuentes Coronel, Luis Hernández, Carmen López Ramos, Laura Mata, Concepción Piñero, Beatriz Sicilia, Mónica Sierra, Mónica Vásquez, Montserrat Aceituno, Michelle Bautista, Xavier Calvet, Maria Esteve Comas, Antonia Montserrat, José Antonio Gómez Valero, Jordi Gordillo, Cesar Ledezma, Laia Lluis, Francisco J. Martínez-Cerezo, Margarita Menacho, Mercè Navarro-Llavat, Silvia Rodríguez Mondéjar, Miriam Sabat, Manuela Josefa Sampedro, Eva Sesé Abizanda, Anibal Silva, Sandra Torra Alsina, Leyanira Torrealba, Carmen Vila Lolo, Marta Ágreda Chinea, Alicia Algaba, Fernando Bermejo, Isabel Blázquez Gómez, Orencio Bosch, Belén Botella, María José Casanova, Carlos Castaño-Milla, María Chaparro, Rocío De Lucas, Mercedes Domínguez-Antonaya, María G. Donday, Almudena Durán, María Luisa Galve, Laura García Ramírez, Ana Garre, Javier P. Gisbert, Iván Guerra, Paloma Jiménez, Beatriz López Cauce, Antonio López-Sanromán, Pilar López Serrano, Ignacio Marín-Jiménez, María Dolores Martín-Arranz, Adrian G. McNicholl, José Antonio Olmos Jerez, Verónica Opio, José Lázaro Pérez Calle, Rocío Plaza Santos, Ángel Ponferrada Díaz, Elena San Miguel, Eugenia Sánchez Rodríguez, Isabel Vera-Mendoza, Mariam Aguas Marífé García-Sepulcre, Ana Gutiérrez, Belén Herreros Martínez, José María Huguet, Nuria Jiménez, Nuria Maroto, Lidia Martí Romero, Miguel Mínguez, Margarita Muñoz Vicente, Pablo Navarro, Pilar Nos, José Joaquín Ramírez Palanca, Marisa Roldán Lafuente, Liliana Pozzati, Pilar Robledo, Manu Barreiro-de Acosta, Iria Bastón-Rey, Amalia Carmona, Daniel Carpio, Elena Castro, Belén Crespo Suarez, María Teresa Diz-Lois Palomares, Ana Echarri, Jesús Daniel Fernández-de Castro, Estela Fernández Salgado, Rocío Ferreiro-Iglesias, Vicent Hernández, Alina López Baz, Pablo Pérez Galindo, María Jesús Ruiz Barcia, Pablo Vega, Margalida Calafat, Daniel Ginard, Eduardo Iyo, María Teresa Novella Durán, Josep Reyes, Carolina Rodríguez Hidalgo, Vanesa Royo, Amparo Sapiña, Ana Belen Aliende, Hipólito Fernández Rosáenz, María Fraile González, Alba García Rodríguez, Berta Lapeña Muñoz, María Teresa Moyano, Susana Revuelta Martínez, Rebeca Irisarri, Marcos Kutz, Óscar Nantes, Cristina Rodríguez, Saioa Rubio, Miren Vicuña, Horacio Alonso, Jose Luis Cabriada, Agustin Castiella Eguzkiza, Itziar Galdona, Ainara Maíz, Ana Isabel Muñagorri Santos, Nerea Muro Carral, Jone Ortiz de Zarate, Nora Otegui, Iago Rodríguez-Lago, Katerina Spicakova, Eva María Zapata, Leire Zubiaurre Lizarralde, Jose Manuel Castillo Espinosa, María del Carmen Martínez Bonil, Cristina Martínez Pascual, Isabel Nicolás and Emilio Torrella Cortés.
