# Correction: Chaparro et al. Incidence, Clinical Characteristics and Management of Inflammatory Bowel Disease in Spain: Large-Scale Epidemiological Study. *J. Clin. Med.* 2021, *10*, 2885

**DOI:** 10.3390/jcm11195816

**Published:** 2022-09-30

**Authors:** María Chaparro, Ana Garre, Andrea Núñez Ortiz, María Teresa Diz-Lois Palomares, Cristina Rodríguez, Sabino Riestra, Milagros Vela, José Manuel Benítez, Estela Fernández Salgado, Eugenia Sánchez Rodríguez, Vicent Hernández, Rocío Ferreiro-Iglesias, Ángel Ponferrada Díaz, Jesús Barrio, José María Huguet, Beatriz Sicilia, María Dolores Martín-Arranz, Xavier Calvet, Daniel Ginard, Inmaculada Alonso-Abreu, Luis Fernández-Salazar, Pilar Varela Trastoy, Montserrat Rivero, Isabel Vera-Mendoza, Pablo Vega, Pablo Navarro, Mónica Sierra, José Luis Cabriada, Mariam Aguas, Raquel Vicente, Mercè Navarro-Llavat, Ana Echarri, Fernando Gomollón, Elena Guerra del Río, Concepción Piñero, María José Casanova, Katerina Spicakova, Jone Ortiz de Zarate, Emilio Torrella Cortés, Ana Gutiérrez, Horacio Alonso-Galán, Álvaro Hernández-Martínez, José Miguel Marrero, Rufo Lorente Poyatos, Margalida Calafat, Lidia Martí Romero, Pilar Robledo, Orencio Bosch, Nuria Jiménez, María Esteve Comas, José María Duque, Ana María Fuentes Coronel, Manuela Josefa Sampedro, Eva Sesé Abizanda, Belén Herreros Martínez, Liliana Pozzati, Hipólito Fernández Rosáenz, Belén Crespo Suarez, Pilar López Serrano, Alfredo J. Lucendo, Margarita Muñoz Vicente, Fernando Bermejo, José Joaquín Ramírez Palanca, Margarita Menacho, Amalia Carmona, Raquel Camargo, Sandra Torra Alsina, Nuria Maroto, Juan Nerín de la Puerta, Elena Castro, Ignacio Marín-Jiménez, Belén Botella, Amparo Sapiña, Noelia Cruz, José Luis F. Forcelledo, Abdel Bouhmidi, Carlos Castaño-Milla, Verónica Opio, Isabel Nicolás, Marcos Kutz, Alfredo Abraldes Bechiarelli, Jordi Gordillo, Yolanda Ber, Yolanda Torres Domínguez, María Teresa Novella Durán, Silvia Rodríguez Mondéjar, Francisco J. Martínez-Cerezo, Lilyan Kolle, Miriam Sabat, Cesar Ledezma, Eduardo Iyo, Óscar Roncero, Rebeca Irisarri, Laia Lluis, Isabel Blázquez Gómez, Eva María Zapata, María José Alcalá, Cristina Martínez Pascual, María Montealegre, Laura Mata, Ana Monrobel, Alejandro Hernández Camba, Luis Hernández, María Tejada, Alberto Mir, María Luisa Galve, Marta Soler, Daniel Hervías, José Antonio Gómez-Valero, Manuel Barreiro-de Acosta, Fernando Rodríguez-Artalejo, Esther García-Esquinas, Javier P. Gisbert, on behalf of the EpidemIBD Study Group of GETECCU

**Affiliations:** 1 Department of Gastroenterology, Hospital Universitario de La Princesa, Instituto de Investigación Sanitaria Princesa (IIS-IP), Universidad Autónoma de Madrid, and Centro de Investigación Biomédica en Red de Enfermedades Hepáticas y Digestivas (CIBERehd), 28006 Madrid, Spain; 2 Department of Gastroenterology, Hospital Universitario Virgen del Rocío, 41013 Sevilla, Spain; 3 Department of Gastroenterology, Hospital Universitario A Coruña, 15006 A Coruña, Spain; 4 Department of Gastroenterology, Complejo Hospitalario de Navarra, 31008 Pamplona, Spain; 5 Department of Gastroenterology, Hospital Universitario Central de Asturias and ISPA, 33011 Oviedo, Spain; 6 Department of Gastroenterology, Complejo Hospitalario Universitario Ntra. Sra. de Candelaria, 38010 Santa Cruz de Tenerife, Spain; 7 Department of Gastroenterology, Hospital Universitario Reina Sofía and IMIBIC, 14004 Córdoba, Spain; 8 Department of Gastroenterology, Complexo Hospitalario Universitario de Pontevedra, Instituto de Investigación Sanitaria Galicia Sur, 36071 Pontevedra, Spain; 9 Department of Gastroenterology, Hospital Ramón y Cajal, 28034 Madrid, Spain; 10 Department of Gastroenterology, Hospital Álvaro Cunqueiro, Estrutura Organizativa de Xestión Integrada de Vigo, 36213 Vigo, Spain; 11 Department of Gastroenterology, Complexo Hospitalario Universitario de Santiago, 15706 Santiago de Compostela, Spain; 12 Department of Gastroenterology, Hospital Universitario Infanta Leonor, 28031 Madrid, Spain; 13 Department of Gastroenterology, Hospital Universitario Rio Hortega, 47012 Valladolid, Spain; 14 Department of Gastroenterology, Consorcio Hospital General Universitario de Valencia, 46014 Valencia, Spain; 15 Department of Gastroenterology, Hospital Universitario de Burgos, 09006 Burgos, Spain; 16 Department of Gastroenterology, Hospital Universitario La Paz, 28046 Madrid, Spain; 17 Servei de Malalties Digestives, Hospital de Sabadell, Institut Universitari Parc Taulí, Universitat Autònoma de Barcelona, CIBEREHD—Instituto de Salud Carlos III, Parc Taulí, 1, 08208 Sabadell, Barcelona, Spain; 18 Department of Gastroenterology, Hospital Universitari Son Espases, 07120 Palma de Mallorca, Spain; 19 Department of Gastroenterology, Hospital Universitario de Canarias (H.U.C.), 38320 Santa Cruz de Tenerife, Spain; 20 Department of Gastroenterology, Hospital Clínico Universitario de Valladolid, 47003 Valladolid, Spain; 21 Department of Gastroenterology, Hospital de Cabueñes, 33394 Gijón, Spain; 22 Department of Gastroenterology, Hospital Universitario Marqués de Valdecilla and IDIVAL, 39008 Santander, Spain; 23 Department of Gastroenterology, Hospital Universitario Puerta de Hierro Majadahonda, 28222 Madrid, Spain; 24 Department of Gastroenterology, Complexo Hospitalario Universitario de Ourense, 32005 Ourense, Spain; 25 Department of Gastroenterology, Hospital Clínico Universitario de Valencia, Universitat de València, 46010 Valencia, Spain; 26 Department of Gastroenterology, Complejo Asistencial Universitario de León, 24001 León, Spain; 27 Department of Gastroenterology, Hospital de Galdakao-Usansolo, Galdakao, 48960 Vizcaya, Spain; 28 Department of Gastroenterology, Hospital Universitari i Politecnic La Fe and CIBERehd, 46026 Valencia, Spain; 29 Department of Gastroenterology, Hospital Universitario Miguel Servet, 50009 Zaragoza, Spain; 30 Department of Gastroenterology, Hospital de Sant Joan Despí Moisès Broggi, 08970 Barcelona, Spain; 31 Department of Gastroenterology, Complejo Hospitalario Universitario de Ferrol, 15405 A Coruña, Spain; 32 Department of Gastroenterology, Hospital Clínico Universitario “Lozano Blesa”, IIS Aragón and CIBERehd, 50009 Zaragoza, Spain; 33 Department of Gastroenterology, Hospital Universitario de Gran Canaria Dr. Negrín, 35010 Las Palmas, Spain; 34 Department of Gastroenterology, Hospital Universitario de Salamanca, 37007 Salamanca, Spain; 35 Department of Gastroenterology, Hospital Universitario de Araba (sede Txagorritxu y sede Santiago), 01009 Álava, Spain; 36 Department of Gastroenterology, Hospital Universitario de Basurto, 48013 Bilbao, Spain; 37 Department of Gastroenterology, Hospital General Universitario J.M. Morales Meseguer, 30008 Murcia, Spain; 38 Department of Gastroenterology, Hospital General Universitario de Alicante and CIBERehd, 03010 Alicante, Spain; 39 Department of Gastroenterology, Hospital Universitario Donostia-Donostia Unibertsitate Ospitalea, Guipuzkoa and Organizacion Sanitaria Integrada Tolosaldea, Clínica Santa María de la Asunción, 20014 Guipúzcoa, Spain; 40 Department of Gastroenterology, Complejo Hospitalario de Especialidades Torrecárdenas, 04009 Almería, Spain; 41 Department of Gastroenterology, Hospital Universitario Insular de Gran Canaria, 35016 Las Palmas, Spain; 42 Department of Gastroenterology, Hospital General Universitario de Ciudad Real, 13005 Ciudad Real, Spain; 43 Department of Gastroenterology, Hospital Son Llatzer, 07198 Palma de Mallorca, Spain; 44 Department of Gastroenterology, Hospital Francesc De Borja de Gandía, 46702 Valencia, Spain; 45 Department of Gastroenterology, Hospital Universitario de Cáceres, 10004 Cáceres, Spain; 46 Department of Gastroenterology, Hospital Universitario Fundación Jiménez Díaz, 28040 Madrid, Spain; 47 Department of Gastroenterology, Hospital General Universitario de Elche, 03203 Alicante, Spain; 48 Department of Gastroenterology, Hospital Universitari Mutua Terrasa, 08221 Terrassa, Spain; 49 Department of Gastroenterology, Hospital San Agustín, 33401 Avilés, Spain; 50 Department of Gastroenterology, Hospital Virgen de La Concha, Complejo Asistencial de Zamora, 49022 Zamora, Spain; 51 Department of Gastroenterology, CSDM, Hospital de Mataró, 08304 Barcelona, Spain; 52 Department of Gastroenterology, Hospital Universitario Arnau de Vilanova, 25198 Lérida, Spain; 53 Department of Gastroenterology, Hospital De Villajoyosa, 03570 Alicante, Spain; 54 Department of Gastroenterology, Hospital de Mérida, 06800 Mérida, Spain; 55 Department of Gastroenterology, Hospital San Pedro, 26006 Logroño, Spain; 56 Department of Gastroenterology, Hospital da Costa (EOXI Lugo-Cervo-Monforte), 27880 Lugo, Spain; 57 Department of Gastroenterology, Hospital Universitario Fundación Alcorcón, 28922 Madrid, Spain; 58 Department of Gastroenterology, Hospital General de Tomelloso, 13700 Tomelloso, Spain; 59 Instituto de Investigación Sanitaria Princesa (IIS-IP), 28006 Madrid, Spain; 60 Centro de Investigación Biomédica en Red de Enfermedades Hepáticas y Digestivas (CIBERehd), 28006 Madrid, Spain; 61 Department of Gastroenterology, Hospital General Universitario de Castellón, 12004 Castellón, Spain; 62 Department of Gastroenterology, Hospital Universitario de Fuenlabrada and Instituto de Investigación Sanitaria Hospital La Paz (IdiPaz), 28942 Madrid, Spain; 63 Department of Gastroenterology, Hospital Lluis Alcanyis, Xátiva, 46800 Valencia, Spain; 64 Department of Gastroenterology, Hospital Joan XXIII, 43005 Tarragona, Spain; 65 Department of Gastroenterology, Hospital Povisa, 36211 Pontevedra, Spain; 66 Department of Gastroenterology, Complejo Hospitalario de Especialidades Virgen de la Victoria, 29010 Málaga, Spain; 67 Department of Gastroenterology, Parc Sanitari Sant Joan de Déu, 08830 Barcelona, Spain; 68 Department of Gastroenterology, Hospital de Manises, 46940 Valencia, Spain; 69 Department of Gastroenterology, Hospital Royo Villanova, 50015 Zaragoza, Spain; 70 Department of Gastroenterology, Complexo Hospitalario Universitario Xeral-Calde de Lugo, 27004 Lugo, Spain; 71 Department of Gastroenterology, Hospital General Universitario Gregorio Marañón, Instituto de Investigación Biomédica Gregorio Marañón (IiSGM), 28007 Madrid, Spain; 72 Department of Gastroenterology, Hospital Universitario Infanta Cristina, 28981 Madrid, Spain; 73 Department of Gastroenterology, Hospital de Manacor, 07500 Manacor, Spain; 74 Department of Gastroenterology, Hospital Doctor José Molina Orosa, 35500 Lanzarote, Spain; 75 Department of Gastroenterology, Hospital Comarcal Sierrallana, 39300 Torrelavega, Spain; 76 Department of Gastroenterology, Hospital Santa Bárbara, 13500 Puertollano, Spain; 77 Department of Gastroenterology, Hospital Rey Juan Carlos, 28933 Madrid, Spain; 78 Department of Gastroenterology, Hospital Universitario de Getafe, 28905 Madrid, Spain; 79 Department of Gastroenterology, Hospital General Universitario Reina Sofía, 30003 Murcia, Spain; 80 Department of Gastroenterology, Hospital Reina Sofía, 31500 Tudela, Spain; 81 Department of Gastroenterology, Hospital Puerta del Mar, 11009 Cádiz, Spain; 82 Department of Gastroenterology, Hospital de la Santa Creu i Sant Pau, 08041 Barcelona, Spain; 83 Department of Gastroenterology, Hospital San Jorge, 22004 Huesca, Spain; 84 Department of Gastroenterology, Hospital San Juan de Dios del Aljarafe, 41930 Sevilla, Spain; 85 Department of Gastroenterology, Hospital Can Misses, 07800 Ibiza, Spain; 86 Department of Gastroenterology, Hospital Sant Joan de Deu, 08950 Barcelona, Spain; 87 Department of Gastroenterology, Hospital Universitari Sant Joan, 25001 Lérida, Spain; 88 Department of Gastroenterology, Hospital General de La Palma, 38713 Santa Cruz de Tenerife, Spain; 89 Department of Gastroenterology, Hospital Santa Caterina, 17190 Gerona, Spain; 90 Department of Gastroenterology, Hospital Palamós, 17230 Girona, Spain; 91 Department of Gastroenterology, Hospital Comarcal de Inca, 07300 Inca, Spain; 92 Department of Gastroenterology, Hospital General La Mancha Centro, 13600 Ciudad Real, Spain; 93 Department of Gastroenterology, Hospital García Orcoyen, 31200 Estella, Spain; 94 Department of Gastroenterology, Hospital Sagrat Cor, 08029 Barcelona, Spain; 95 Department of Gastroenterology, Hospital de Torrejón, 28850 Madrid, Spain; 96 Department of Gastroenterology, Hospital de Mendaro, 20850 Guipuzkoa, Spain; 97 Department of Gastroenterology, Hospital Obispo Polanco, 44002 Teruel, Spain; 98 Department of Gastroenterology, Hospital General Universitario Los Arcos del Mar Menor, San Javier, 30739 Murcia, Spain; 99 Department of Gastroenterology, Hospital General de Villarobledo, 02600 Albacete, Spain; 100 Department of Gastroenterology, Hospital Medina del Campo, 47400 Valladolid, Spain; 101 Department of Gastroenterology, Hospital de Montilla, 14550 Córdoba, Spain; 102 Department of Gastroenterology, Hospital Quirón Costa Adeje, 38660 Santa Cruz de Tenerife, Spain; 103 Department of Gastroenterology, Hospital Santos Reyes, 09400 Aranda de Duero, Spain; 104 Department of Gastroenterology, Clínica Astarté, 11011 Cádiz, Spain; 105 Department of Gastroenterology, Hospital Ernest Lluch, 50299 Zaragoza, Spain; 106 Department of Gastroenterology, Hospital Central de La Cruz Roja San José y Santa Adela, 28003 Madrid, Spain; 107 Department of Gastroenterology, Hospital San Juan De Dios, 38009 Tenerife, Spain; 108 Department of Gastroenterology, Hospital Virgen de Altagracia, 13002 Manzanares, Spain; 109 Department of Gastroenterology, Hospital Dexeus, 08028 Barcelona, Spain; 110 Department of Preventive Medicine and Public Health, School of Medicine, Universidad Autónoma de Madrid/IdiPaz and CIBERESP, 28029 Madrid, Spain

The authors wish to make the following corrections to this paper [1]. On page 18 of the published paper (backmatter, Acknowledgments), the authors would like to complete the following inaccurate paragraph:

**Acknowledgments:** The authors wish to express thanks to Almudena Durán and Paloma Jiménez for database monitoring and to Guillermo Ortega and Ancor Sanz for hospital clustering.

With the following sentence:

**Acknowledgments:** The authors wish to express thanks to Almudena Durán and Paloma Jiménez for database monitoring and to Guillermo Ortega and Ancor Sanz for hospital clustering. The authors also wish to thank the members of Grupo EpidemIBD.

In addition, add the Appendix section:

## Appendix A

**The EpidemIBD Study Group Of Geteccu:**

Alfredo Abraldes Bechiarelli, José Manuel Benítez, María del Rosario Calderón, Raquel Camargo, Álvaro Hernández-Martínez, Eva Iglesias Flores, Eduardo Leo Carnerero, Sandra Marín Pedrosa, Ana Monrobel, Andrea Núñez Ortiz, Natalia Ruiz Santana, María Tejada, Yolanda Torres Domínguez, María José Alcalá, Erika Alfambra, Yolanda Ber, Fernando Gomollón, Miguel Montoro, Juan Manuel Nerín, Elena Peña, Raquel Vicente, José María Duque, Ruth de Francisco, Alejo Mancebo Mata, Susana Martínez González, Isabel Pérez Martínez, Sabino Riestra, Pilar Varela Trastoy, Inmaculada Alonso-Abreu, Daniel Ceballos, Noelia Cruz, Elena Guerra del Río, Alejandro Hernández Camba, Lilian Kole, José Miguel Marrero, Nuria Pérez, Marta Soler, Milagros Vela, José Luis F. Forcelledo, Montserrat Rivero, Rufo Lorente Poyatos, Cristina Verdejo Gil, María Montealegre, Alfredo J. Lucendo, Óscar Roncero, Abdel Bouhmidi, Daniel Hervías, Lara Arias García, Jesús Barrio, Ana Y. Carbajo, Luis Fernández-Salazar, Paola Fradejas, Ana María Fuentes Coronel, Luis Hernández, Carmen López Ramos, Laura Mata, Concepción Piñero, Beatriz Sicilia, Mónica Sierra, Mónica Vásquez, Montserrat Aceituno, Michelle Bautista, Xavier Calvet, Maria Esteve Comas, Antonia Montserrat, José Antonio Gómez Valero, Jordi Gordillo, Cesar Ledezma, Laia Lluis, Francisco J. Martínez-Cerezo, Margarita Menacho, Mercè Navarro-Llavat, Silvia Rodríguez Mondéjar, Miriam Sabat, Manuela Josefa Sampedro, Eva Sesé Abizanda, Anibal Silva, Sandra Torra Alsina, Leyanira Torrealba, Carmen Vila Lolo, Marta Ágreda Chinea, Alicia Algaba, Fernando Bermejo, Isabel Blázquez Gómez, Orencio Bosch, Belén Botella, María José Casanova, Carlos Castaño-Milla, María Chaparro, Rocío De Lucas, Mercedes Domínguez-Antonaya, María G. Donday, Almudena Durán, María Luisa Galve, Laura García Ramírez, Ana Garre, Javier P. Gisbert, Iván Guerra, Paloma Jiménez, Beatriz López Cauce, Antonio López-Sanromán, Pilar López Serrano, Ignacio Marín-Jiménez, María Dolores Martín-Arranz, Adrian G. McNicholl, José Antonio Olmos Jerez, Verónica Opio, José Lázaro Pérez Calle, Rocío Plaza Santos, Ángel Ponferrada Díaz, Elena San Miguel, Eugenia Sánchez Rodríguez, Isabel Vera-Mendoza, Mariam Aguas Marífé García-Sepulcre, Ana Gutiérrez, Belén Herreros Martínez, José María Huguet, Nuria Jiménez, Nuria Maroto, Lidia Martí Romero, Miguel Mínguez, Margarita Muñoz Vicente, Pablo Navarro, Pilar Nos, José Joaquín Ramírez Palanca, Marisa Roldán Lafuente, Liliana Pozzati, Pilar Robledo, Manu Barreiro-de Acosta, Iria Bastón-Rey, Amalia Carmona, Daniel Carpio, Elena Castro, Belén Crespo Suarez, María Teresa Diz-Lois Palomares, Ana Echarri, Jesús Daniel Fernández-de Castro, Estela Fernández Salgado, Rocío Ferreiro-Iglesias, Vicent Hernández, Alina López Baz, Pablo Pérez Galindo, María Jesús Ruiz Barcia, Pablo Vega, Margalida Calafat, Daniel Ginard, Eduardo Iyo, María Teresa Novella Durán, Josep Reyes, Carolina Rodríguez Hidalgo, Vanesa Royo, Amparo Sapiña, Ana Belen Aliende, Hipólito Fernández Rosáenz, María Fraile González, Alba García Rodríguez, Berta Lapeña Muñoz, María Teresa Moyano, Susana Revuelta Martínez, Rebeca Irisarri, Marcos Kutz, Óscar Nantes, Cristina Rodríguez, Saioa Rubio, Miren Vicuña, Horacio Alonso, Jose Luis Cabriada, Agustin Castiella Eguzkiza, Itziar Galdona, Ainara Maíz, Ana Isabel Muñagorri Santos, Nerea Muro Carral, Jone Ortiz de Zarate, Nora Otegui, Iago Rodríguez-Lago, Katerina Spicakova, Eva María Zapata, Leire Zubiaurre Lizarralde, Jose Manuel Castillo Espinosa, María del Carmen Martínez Bonil, Cristina Martínez Pascual, Isabel Nicolás and Emilio Torrella Cortés.

The authors apologize for any inconvenience caused and state that the scientific conclusions are unaffected. The original article was updated.
