# Supplementary material for: Incidence, Clinical Characteristics and Management of Inflammatory Bowel Disease in Spain: Large-Scale Epidemiological Study
Source: J Clin Med. 2021 Jun 29;10(13):2885. doi: 10.3390/jcm10132885 (PMC8268420; doi:10.3390/jcm10132885)
Supplement: Supplementary file 1 [file jcm-10-02885-s001.zip › jcm-1246641-supplementary.pdf]

Supplementary Table S1. Incidence rates (cases/100,000 person-years) for inflammatory bowel disease by Autonomous Community in Spain, 2017.

Autonomous Community	Number of centers	Number of cases	Incidence rates
Andalucía	8	280	11.76
Aragón	6	146	13.49
Asturias	3	167	21.18
Baleares	5	138	13.92
Canarias	8	252	13.77
Cantabria	2	73	15.48
Castilla La Mancha	6	106	18.05
Castilla y León	8	321	16.21
Cataluña	14	336	11.23
Comunidad Valenciana	10	371	16.29
Extremadura	2	61	16.64
Galicia	9	472	16.51
Madrid	14	478	12.35
Murcia	3	70	12.39
Navarra	3	119	19.04
País Vasco	6	192	13.28
La Rioja	1	29	9.64
TOTAL	108	3,611	16.2

Supplementary Table S2. Extraintestinal manifestations at diagnosis in the inception cohort.

Peripheral arthropathy, n (%)	140 (4)
Cutaneous, n (%)	64 (1.8)
Spondiloarthritis, n (%)	47 (1.3)
Estomatitis, n (%)	35 (1)
Ophthalmological, n (%)	26 (0.7)
Sacroilitis, n (%)	22 (0.6)
Primary sclerosing cholangitis, n (%)	9 (0.2)
Other, n (%)	46 (1.3)
Overall patients with at least one extraintestinal manifestation, n (%)	327 (9)

Supplementary Table S3. Examinations performed at inflammatory bowel disease diagnosis. Examinations performed at inflammatory bowel disease diagnosis.

Colonoscopy, n (%)	3,563 (99)
Abdominal CT scan, n (%)	627 (17.4)
MR enterography, n (%)	567 (15.8)
Upper gastrointestinal endoscopy, n (%)	264 (7.3)
Abdominal ultrasound, n (%)	163 (4.5)
Capsule endoscopy, n (%)	103 (2.9)
Pelvic MR, n (%)	76 (2.1)
Small bowel follow-through, n (%)	56 (1.6)
Endoanal ultrasound, n (%)	26 (0.7)
Fistula examination under anesthesia, n (%)	23 (0.6)

Supplementary Table S4. Reasons for hospitalizations in the first year after diagnosis.

Disease debuts , n (%)	731 (20)
Disease flares, n (%)	130 (3.6)
Intestinal obstructions, n (%)	42 (1.2)
Adverse reactions to treatments, n (%)	27 (0.7)
Perianal disease, n (%)	23 (0.6)
Abdominal abscesses, n (%)	14 (0.4)
Elective surgeries, n (%)	14 (0.4)
Infections, n (%)	9 (0.3)
Intestinal perforations, n (%)	9 (0.3)
Overall, n (%)	1,012 (28)

Supplementary Table S5. Patients' characteristics based on hospital categories.

	Category 1<br>N=18	Category 2<br>N=159	Category 3<br>N=921	Category 4<br>N=1,039	Category 5<br>N=1,474	p
Age, years (median, IQR)	48 (36-55)	44 (31-56)	44 (33-55)	42 (31-56)	44 (31-56)	>0.05
Male gender, n (%)	10 (55.6)	91 (57)	504 (55)	548 (53)	755 (51)	>0.05
Former smokers, n (%)	4 (23.5)	38 (24)	235 (25.5)	253 (24.5)	350 (24)	>0.05
Symptoms at diagnosis, n (%)	18 (100)	148 (94)	850 (92.6)	937 (91)	1,327 (91.3)	>0.05
Diagnostic delay, months (median, IQR)	1.5 (0-4.2)	5 (2-18)	3 (1-7)	3 (1-9)	3 (1-9)	<0.05
Time from symptoms onset to primary care consultation, months (median, IQR)	2 (0.7-3.7)	0 (0-2)	0 (0-2)	0 (0-1)	0 (0-2)	<0.05
Time from primary care to gastroenterologist consultation, months (median, IQR)	1 (0-2)	2 (0-4)	1 (0-4)	2 (0-4)	2 (1-5)	<0.05
Family history of IBD, n (%)	0 (0)	24 (15)	127 (14)	137 (13.4)	236 (16)	>0.05
Extraintestinal manifestations, n (%)	2 (11)	21 (13)	60 (6.5)	84 (8)	160 (11)	>0.05
Crohn's disease, n (%)	7 (39)	81 (51)	409 (44.5)	453 (43.6)	697 (47)	>0.05
Ulcerative colitis, n (%)	9 (50)	73 (46)	464 (50.4)	552 (53)	709 (48)	>0.05
Unclassified inflammatory bowel disease, n (%)	2 (11)	5 (3)	47 (5)	34 (3.3)	68 (4.6)	>0.05
Mesalamine ever, n (%)	15 (83)	121 (76)	598 (65)	728 (70)	988 (67)	<0.01
Steroids ever, n (%)	6 (33)	96 (60.4)	477 (52)	532 (51)	805 (54.6)	<0.05
Systemic steroid therapy, n (%)	4 (22)	49 (31)	306 (33)	371 (36)	522 (35.4)	>0.05
Immunomodulators, n (%)	2 (11)	54 (34)	245 (26.6)	267 (26)	368 (25)	>0.05
Biologics, n (%)	1 (5.6)	21 (13)	140 (15)	162 (15.6)	234 (16)	>0.05
Surgeries, n (%)	0 (0)	8 (5)	42 (4.6)	50 (4.8)	99 (6.7)	>0.05
Hospitalizations, n (%)	2 (11)	37 (23)	236 (25.6)	324 (31)	413 (28)	<0.05

Categories, 1: small general hospitals, 2: medium general hospitals, 3: general hospitals, 4: referral hospitals, 5: large referral hospitals.

Grupo EpidemIBD

ANDALUCÍA:

Alfredo Abraldes Bechiarelli. Hospital Puerta del Mar, Cádiz
José Manuel Benítez. Hospital Universitario Reina Sofía and IMIBIC, Córdoba (Coordinador Andalucía)
María del Rosario Calderón. Clínica Astarté, Cádiz.
Raquel Camargo. Complejo Hospitalario de Especialidades Virgen de la Victoria, Málaga
Álvaro Hernández-Martínez. Complejo Hospitalario de Especialidades Torrecárdenas, Almería.
Eva Iglesias Flores. Hospital Universitario Reina Sofía and IMIBIC, Córdoba
Eduardo Leo Carnerero. Hospital Universitario Virgen del Rocío, Sevilla
Sandra Marín Pedrosa. Hospital Universitario Reina Sofía and IMIBIC, Córdoba
Ana Monrobel. Hospital de Montilla, Córdoba
Andrea Núñez Ortiz. Hospital Universitario Virgen del Rocío, Sevilla
Natalia Ruiz Santana. Complejo Hospitalario de Especialidades Virgen de la Victoria, Málaga.
María Tejada. Clínica Astarté, Cádiz
Yolanda Torres Domínguez. Hospital San Juan de Dios del Aljarafe, Sevilla

ARAGÓN:

María José Alcalá. Hospital Obispo Polanco, Teruel
Erika Alfambra. Hospital Lozano Blesa, Zaragoza
Yolanda Ber. Hospital San Jorge, Huesca
Fernando Gomollón, Hospital Clínico Universitario "Lozano Blesa", IIS Aragón y CIBERehd, Zaragoza. (Coordinador Aragón)
Miguel Montoro. Hospital San Jorge, Huesca
Juan Manuel Nerín. Hospital Royo Villanova, Zaragoza
Elena Peña. Hospital Royo Villanova, Zaragoza
Raquel Vicente. Hospital Universitario Miguel Servet, Zaragoza

ASTURIAS:

José María Duque. Hospital San Agustín, Avilés
Ruth de Francisco. Hospital Universitario Central de Asturias, Oviedo
Alejo Mancebo Mata. Hospital de Cabueñes, Gijón
Susana Martínez González. Hospital Universitario Central de Asturias, Oviedo
Isabel Pérez Martínez. Hospital Universitario Central de Asturias, Oviedo
Sabino Riestra. Hospital Universitario Central de Asturias e ISPA, Oviedo (Coordinador Asturias)
Pilar Varela Trastoy. Hospital de Cabueñes, Gijón

CANARIAS:

Inmaculada Alonso-Abreu. Hospital Universitario de Canarias (H.U.C), Santa Cruz de Tenerife
Daniel Ceballos. Hospital Universitario de Gran Canaria Dr. Negrin, Las Palmas (Coordinador Canarias)
Noelia Cruz. Hospital Doctor José Molina Orosa, Lanzarote
Elena Guerra del Río. Hospital Universitario de Gran Canaria Dr. Negrín, Las Palmas
Alejandro Hernández Camba. Hospital Quirón Costa Adeje, Santa Cruz de Tenerife
Lilian Kole. Hospital General de La Palma, Santa Cruz de Tenerife
José Miguel Marrero. Hospital Universitario Insular de Gran Canaria, Las Palmas
Nuria Pérez. Hospital Universitario de Gran Canaria Dr. Negrin, Las Palmas
Marta Soler. Hospital San Juan De Dios, Tenerife
Milagros Vela. Complejo Hospitalario Universitario Ntra. Sra. de Candelaria, Santa Cruz de Tenerife

CANTABRIA:

José Luis F Forcelledo. Hospital Comarcal Sierrallana, Torrelavega
Montserrat Rivero. Hospital Universitario Marqués de Valdecilla, Santander and IDIVAL (Coordinadora Cantabria)

CASTILLA LA MANCHA:

Rufo Lorente Poyatos. Hospital General Universitario de Ciudad Real, Ciudad Real. (Coordinador Castilla La Mancha)
Cristina Verdejo Gil. Hospital General Universitario de Ciudad Real, Ciudad Real
María Montealegre. Hospital General de Villarobledo, Albacete
Alfredo J Lucendo. Hospital General de Tomelloso, Instituto de Investigación Sanitaria Princesa (IIS-IP), and Centro de Investigación Biomédica en Red de Enfermedades Hepáticas y Digestivas (CIBERehd), Tomelloso
Óscar Roncero. Hospital General La Mancha Centro, Ciudad Real
Abdel Bouhmidi. Hospital Santa Bárbara, Puertollano
Daniel Hervías. Hospital Virgen de Altagracia, Manzanares

CASTILLA LEÓN

Lara Arias García. Hospital Universitario de Burgos, Burgos
Jesús Barrio. Hospital Universitario Rio Hortega, Valladolid
Ana Y Carbajo. Hospital Universitario Rio Hortega, Valladolid
Luis Fernández-Salazar Hospital Clínico Universitario de Valladolid, Valladolid
Paola Fradejas. Hospital Virgen de La Concha, Zamora
Ana María Fuentes Coronel. Hospital Virgen de La Concha, Complejo Asistencial de Zamora, Zamora
Luis Hernández. Hospital Santos Reyes, Aranda de Duero
Carmen López Ramos. Hospital Virgen de La Concha, Complejo Asistencial de Zamora, Zamora
Laura Mata. Hospital Medina del Campo, Valladolid
Concepción Piñero. Hospital Universitario de Salamanca, Salamanca
Beatriz Sicilia. Hospital Universitario de Burgos, Burgos (Coordinadora Castilla León)
Mónica Sierra. Complejo Asistencial Universitario de León, León
Mónica Vásquez. Hospital Santos Reyes, Aranda de Duero

CATALUÑA

Montserrat Aceituno. Hospital Universitari Mutua Terrasa, Terrassa
Michelle Bautista. Hospital Universitario San Joan de Reus, Tarragona
Xavier Calvet. Hospital Universitari Parc Taulí, Sabadell. Departament de Medicina, Universitat Autònoma de Barcelona. CIBERehd - Instituto de Salud Carlos III, Barcelona
Maria Esteve Comas. Hospital Universitari Mutua Terrasa, Terrassa (Coordinadora Cataluña)
Antonia Montserrat. Hospital Universitari Parc Taulí, Sabadell.
José Antonio Gómez Valero. Hospital Dexeus, Grupo Quirónsalud, Barcelona
Jordi Gordillo. Hospital de la Santa Creu i Sant Pau, Barcelona
Cesar Ledezma. Hospital Palamós, Girona
Laia Lluis. Hospital Sagrat Cor, Barcelona
Francisco J. Martínez-Cerezo. Hospital Universitari Sant Joan, Lérida
Margarita Menacho. Hospital Joan XXIII. Tarragona
Mercè Navarro-Llavat. Hospital de Sant Joan Despí Moisès Broggi, Barcelona
Silvia Rodríguez Mondéjar. Hospital Sant Joan de Deu, Barcelona
Miriam Sabat. Hospital Santa Caterina, Gerona
Manuela Josefa Sampedro. CSDM. Hospital de Mataró, Barcelona.
Eva Sesé Abizanda. Hospital Universitario Arnau de Vilanova, Lérida
Anibal Silva. Parc Sanitari Sant Joan de Déu, Barcelona
Sandra Torra Alsina. Parc Sanitari Sant Joan de Déu, Barcelona
Leyanira Torrealba. Hospital Dr. Josep Trueta, Gerona
Carmen Vila Lolo. Hospital Dexeus, Barcelona

COMUNIDAD DE MADRID

Marta Ágreda Chinea. Hospital Universitario Fundación Jiménez Díaz; Madrid
Alicia Algaba: Hospital Universitario de Fuenlabrada and Instituto de Investigación Sanitaria Hospital La Paz (IdiPaz), Madrid
Fernando Bermejo. Hospital Universitario de Fuenlabrada and Instituto de Investigación Sanitaria Hospital La Paz (IdiPaz), Madrid

Isabel Blázquez Gómez. Hospital de Torrejón, Madrid
Orencio Bosch. Hospital Universitario Fundación Jiménez Díaz; Madrid
Belén Botella. Hospital Universitario Infanta Cristina; Madrid
María José Casanova. Hospital Universitario de La Princesa, IIS-IP y CIBERehd, Madrid (Coordinadora Madrid)
Carlos Castaño-Milla. Hospital Rey Juan Carlos, Madrid
María Chaparro. Hospital Universitario de La Princesa, IIS-IP y CIBERehd, Madrid
Rocío De Lucas. Hospital Universitario Puerta de Hierro Majadahonda, Madrid
Mercedes Domínguez-Antonaya. Hospital Rey Juan Carlos, Madrid
María G Donday. Hospital Universitario de La Princesa, IIS-IP y CIBERehd, Madrid
Almudena Durán. Hospital Universitario de La Princesa, IIS-IP y CIBERehd, Madrid
María Luisa Galve. Hospital Central de La Cruz Roja San José y Santa Adela, Madrid
Laura García Ramírez. Hospital Universitario La Paz, Madrid
Ana Garre. Hospital Universitario de La Princesa, IIS-IP y CIBERehd, Madrid
Javier P. Gisbert. Hospital Universitario de La Princesa, IIS-IP y CIBERehd, Madrid
Iván Guerra. Hospital Universitario de Fuenlabrada, Madrid
Paloma Jiménez. Hospital Universitario de La Princesa, IIS-IP y CIBERehd, Madrid
Beatriz López Cauce. Complejo Hospitalario Gregorio Marañón, Madrid
Antonio López-Sanromán. Hospital Ramón y Cajal, Madrid
Pilar López Serrano. Hospital Universitario Fundación Alcorcón; Madrid
Ignacio Marín-Jiménez. Hospital General Universitario Gregorio Marañón, Instituto de Investigación Biomédica Gregorio Marañón (IiSGM), Madrid
María Dolores Martín-Arranz. Hospital Universitario La Paz, Madrid
Adrian G McNicholl. Hospital Universitario de La Princesa, IIS-IP y CIBERehd, Madrid
José Antonio Olmos Jerez. Hospital Rey Juan Carlos, Madrid
Verónica Opio. Hospital Universitario de Getafe, Madrid
José Lázaro Pérez Calle. Hospital Universitario Fundación Alcorcón; Madrid
Rocío Plaza Santos. Hospital Universitario Infanta Leonor, Madrid
Ángel Ponferrada Díaz. Hospital Universitario Infanta Leonor, Madrid
Elena San Miguel. Hospital Universitario de Getafe, Madrid
Eugenia Sánchez Rodríguez. Hospital Ramón y Cajal, Madrid
Isabel Vera-Mendoza. Hospital Universitario Puerta de Hierro Majadahonda, Madrid
COMUNIDAD VALENCIANA
Mariam Aguas. Hospital Universitari i Politecnic La Fe y CIBERehd, Valencia
Marífé García-Sepulcre. Hospital General Universitario de Elche, Alicante
Ana Gutiérrez. Hospital General Universitario de Alicante y CIBERehd, Alicante
Belén Herreros Martínez. Hospital De Villajoyosa, Alicante
José María Huguet. Consorcio Hospital General Universitario de Valencia, Valencia
Nuria Jiménez. Hospital General Universitario de Elche, Alicante
Nuria Maroto. Hospital de Manises, Valencia
Lidia Martí Romero. Hospital Francesc De Borja de Gandía, Valencia
Miguel Mínguez. Hospital Clínico Universitario de Valencia, Universitat de València
Margarita Muñoz Vicente. Hospital General Universitario De Castellón, Castellón
Pablo Navarro. Hospital Clínico Universitario de Valencia, Universitat de València
Pilar Nos. Hospital Universitari i Politecnic La Fe y CIBERehd, Valencia (Coordinadora Comunidad Valenciana)
José Joaquín Ramírez Palanca. Hospital Lluis Alcanyis, Xátiva, Valencia
Marisa Roldán Lafuente. Hospital General Universitario De Castellón, Castellón
EXTREMADURA
Liliana Pozzati. Hospital de Mérida, Mérida
Pilar Robledo. Hospital Universitario San Pedro de Alcántara, Cáceres (Coordinadora Extremadura)

GALICIA
Manu Barreiro-de Acosta. Complexo Hospitalario Universitario de Santiago, Santiago de Compostela. (Coordinador Galicia)
Iria Bastón -Rey. Complexo Hospitalario Universitario de Santiago, Santiago de Compostela
Amalia Carmona. Hospital Povisa, Pontevedra
Daniel Carpio. Complexo Hospitalario Universitario de Pontevedra, Instituto de Investigación Sanitaria Galicia Sur. Pontevedra
Elena Castro. Complexo Hospitalario Universitario Xeral-Calde de Lugo, Lugo
Belén Crespo Suarez. Hospital da Costa (EOXI Lugo-Cervo-Monforte), Lugo
María Teresa Diz-Lois Palomares. Hospital Universitario A Coruña, A Coruña
Ana Echarri. Complejo Hospitalario Universitario de Ferrol, A Coruña
Jesús Daniel Fernández-de Castro. Complexo Hospitalario Universitario De Ourense, Ourense
Estela Fernández Salgado. Complexo Hospitalario Universitario de Pontevedra, Pontevedra
Rocío Ferreiro-Iglesias. Complexo Hospitalario Universitario de Santiago, Santiago de Compostela
Vicent Hernández. Hospital Álvaro Cunqueiro. Estrutura Organizativa de Xestión Integrada de Vigo, Vigo
Alina López Baz. Complexo Hospitalario Universitario Xeral-Calde de Lugo, Lugo
Pablo Pérez Galindo. Complexo Hospitalario Universitario de Pontevedra, Pontevedra
María Jesús Ruiz Barcia. Hospital da Costa (EOXI Lugo-Cervo-Monforte), Lugo
Pablo Vega. Complexo Hospitalario Universitario De Ourense, Ourense
ISLAS BALEARES:
Margalida Calafat. Hospital Son Llatzer, Palma de Mallorca
Daniel Ginard. Hospital Universitari Son Espases, Palma de Mallorca (Coordinador Islas Baleares)
Eduardo Iyo. Hospital Comarcal de Inca, Inca
María Teresa Novella Durán. Hospital Can Misses, Ibiza
Josep Reyes. Fundación Hospital Comarcal de Inca, Inca
Carolina Rodríguez Hidalgo. Hospital Can Misses, Ibiza
Vanesa Royo. Hospital Universitari Son Espases, Palma de Mallorca
Amparo Sapiña. Hospital de Manacor, Manacor
LA RIOJA
Ana Belen Aliende. Hospital Fundación de Calahorra. La Rioja
Hipólito Fernández Rosáenz. Hospital San Pedro, Logroño. (Coordinador La Rioja)
María Fraile González. Hospital San Pedro, Logroño
Alba García Rodríguez, Hospital San Pedro, Logroño
Berta Lapeña Muñoz. Hospital San Pedro, Logroño.
María Teresa Moyano Matute de Hospital Fundación de Calahorra. La Rioja
Susana Revuelta Martínez, Hospital San Pedro, Logroño.

NAVARRA
Rebeca Irisarri. Hospital García Orcoyen, Estella
Marcos Kutz. Hospital Reina Sofía, Tudela
Óscar Nantes. Complejo Hospitalario De Navarra, Pamplona
Cristina Rodríguez. Complejo Hospitalario De Navarra, Pamplona (Coordinadora Navarra)
Saioa Rubio. Complejo Hospitalario De Navarra, Pamplona
Miren Vicuña. Complejo Hospitalario De Navarra, Pamplona
PAÍS VASCO
Horacio Alonso. Hospital Universitario Donostia-Donostia Unibertsitate Ospitalea, Guipuzkoa.
Jose Luis Cabriada. H. de Galdakao-Usansolo, Galdakao, Vizcaya. (Coordinador País Vasco)
Agustin Castiella Eguzkiza. Hospital de Mendaro, Guipuzkoa
Itziar Galdona. Hospital de Galdakao-Usansolo, Usansolo
Ainara Maíz. Hospital Universitario Donostia-Donostia Unibertsitate Ospitalea, Guipuzkoa
Ana Isabel Muñagorri Santos. Hospital Universitario Donostia-Donostia Unibertsitate Ospitalea, Guipuzkoa

Nerea Muro Carral. Hospital Universitario Donostia-Donostia Unibertsitate Ospitalea, Guipuzkoa
Jone Ortiz de Zarate. Hospital Universitario de Basurto, Bilbao
Nora Otegui. Hospital de Mendaro, Guipuzkoa
Iago Rodríguez-Lago. Hospital Galdakao-Usansolo
Katerina Spicakova. Hospital Universitario de Araba (sede Txagorritxu y sede Santiago), Álava
Eva María Zapata. Hospital de Mendaro, Guipuzkoa
Leire Zubiaurre Lizarralde. Hospital de Mendaro, Guipuzkoa

REGIÓN DE MURCIA

Jose Manuel Castillo Espinosa. Hospital General Universitario Los Arcos del Mar Menor, San Javier, Murcia
María del Carmen Martínez Bonil. Hospital General Universitario Los Arcos del Mar Menor, San Javier, Murcia
Cristina Martínez Pascual, Hospital General Universitario Los Arcos del Mar Menor, San Javier, Murcia
Isabel Nicolás. Hospital General Universitario Reina Sofía; Murcia
Emilio Torrella Cortés. Hospital General Universitario J.M. Morales Meseguer, Murcia (Coordinador Región de Murcia)
